# Rootstock-mediated carbohydrate metabolism, nutrient contents, and physiological modifications in regular and alternate mango (*Mangifera indica* L.) scion varieties

**DOI:** 10.1371/journal.pone.0284910

**Published:** 2023-05-03

**Authors:** Hatkari Vittal, Nimisha Sharma, Anil Kumar Dubey, Mukesh Shivran, Sanjay Kumar Singh, Mahesh Chand Meena, Nirmal Kumar, Neha Sharma, Nisha Singh, Rakesh Pandey, Haritha Bollinedi, Bikram Pratap Singh, Radha Mohan Sharma

**Affiliations:** 1 ICAR-Indian Agricultural Research Institute, New Delhi, India; 2 ICAR-Indian Institute of Horticultural Research, Bengaluru, Karnataka, India; 3 ICAR-National Bureau of Soil Survey & Land Use Planning, Nagpur, Maharashtra, India; 4 IILM- IILM Academy of Higher Learning, College of Engineering and Technology, Greater Noida, Uttar Pradesh, India; 5 Gujarat Biotechnology University, Gandhinagar, Gujarat, India; 6 NIPB-National Institute for Plant Biotechnology, New Delhi, India; United Arab Emirates University, UNITED ARAB EMIRATES

## Abstract

Most of the popular scion varieties of mango possess alternate/irregular bearing. There are many external and internal factors assigned, among them carbohydrate reserves, and nutrient content plays important roles in the floral induction process in many crop species. In addition to that rootstock can alter the carbohydrate reserve and nutrient acquisition of scion varieties in fruit crops. The present investigation was carried out to understand the effect of rootstocks on the physiochemical traits of leaf, and bud and nutrient content in regular and alternate bearing varieties of mango. The rootstock “Kurukkan” promoted starch content in leaves of both alternate bearing varieties ‘Dashehari’ (5.62 mg/g) and regular ‘Amrapali’ (5.49 mg/g) and encouraged higher protein content (6.71 mg/g) and C/N ratio (37.94) in buds of alternate bearing ‘Dashehari’. While Olour rootstock upregulated the reducing sugar in leaves of ‘Amrapali’ (43.56 mg/g) and promoted K (1.34%) and B (78.58 ppm) content in reproductive buds of ‘Dashehari’. Stomatal density in ‘Dashehari’ scion variety was found higher on Olour rootstock (700.40/mm 2), while the rootstock fails to modify stomatal density in the scion variety regular bearer ‘Amrapali’. Further, a total of 30 carbohydrate metabolism-specific primers were designed and validated in 15 scion/rootstock combinations. A total of 33 alleles were amplified among carbohydrate metabolism-specific markers, which varied from 2 to 3 alleles with a mean of 2.53 per locus. Maximum and minimum PIC value was found for NMSPS10, and NMTPS9 primers (0.58). Cluster analysis revealed that scion grafted on Kurukkan rootstock clustered together except ‘Pusa Arunima’ on Olour rootstock. Our analysis revealed that Fe is the key component that is commonly expressed in both leaf and bud. Although Stomatal density (SD) and Intercellular CO_2_ Concentration (Ci) are more specific to leaf and Fe, B, and total sugar (TS) are abundant in buds. Based on the results it can be inferred that the physiochemical and nutrient responses of mango scion varieties are manipulated by the rootstock, hence, the scion-rootstock combination can be an important consideration in mango for selecting suitable rootstock for alternate/irregular bearer varieties.

## Introduction

Mango (*Mangifera indica* L.) is an important commercial fruit crop grown in tropical and sub-tropical parts of the world [1]. Many research studies indicate mango possesses antidiabetic, antioxidant, anti-viral, cardiotonic, hypotensive, and anti-inflammatory properties [2]. It occupies the highest area of 2,315 thousand ha among fruit crops and contributes 20, 899 thousand metric tons of fruit production [3]. Inconsistent yield patterns or irregular bearing are the most frequent situations facing mango production all over the world [4, 5]. Regular flowering is the core event to regulate the bearing behavior of perennial fruit crops. Among intrinsic factors like carbohydrate metabolism, phytohormones and mineral nutrients play a key role in flowering. Carbohydrates reserves depicted as key energy-producing chemicals play important role in the floral induction process in many crop species. The role of carbohydrates in fruit crops is well-studied at the flowering stage [6, 7]. Regulation by carbohydrates is brought about by the accumulation of photo assimilates and their redistribution during each annual production cycle. Draining out of carbohydrate and nitrogen reserves during the “On” year is known to lead to a lean crop in the “Off” year as they are important for fruit bud initiation [8, 9]. The developing fruit provides a strong sink for photo assimilates. This is well supported by the fact that a high endogenous ratio of carbon to nitrogen in plants is stimulatory to flowering whereas a low C: N ratio favors vegetative growth [10]. Nutrient status is an important factor for mango flowering. Recommendations can be made by analyzing the nutrient content of leaves [11]. Zuazo and Tarifa, [12] revealed a direct correlation between mango leaf nutritional content and fruit yield for increasing mango productivity. With the intensification of fruit production due to socioeconomic factors, the use of rootstocks in commercial fruit production has grown significantly in recent years. Research on mango rootstocks has great potential for improvement in mango cultivation. The influence of mango rootstocks on desired characteristics such as tolerance to various biotic and abiotic stress was reported all over the world in different research experiments [13–15]. Rootstocks play a significant role in the process of fruit-bearing behavior, and they may also influence fruit-bearing via physiological parameters that favor production, such as the amount of photosynthate translocated into root portions [16]. Biennial bearing in apples has been found to be reduced by weak or dwarfing rootstocks [17]. Information regarding how rootstock affects the bearing habit of commercial mango varieties is meager. Some studies have found that mango rootstocks have a direct impact on tree growth, flowering, and fruit yield [18, 19]. Both physiological and molecular approaches are important to decipher the role of carbohydrates and nutrients on flowering in alternate and regular-bearing fruit crop varieties. Improving the bearing habit of mango crops requires a thorough understanding of the role of rootstock in carbohydrate metabolism and nutrient composition at physiological and molecular levels, in regular (bearing crop every year) and alternate bearing (bearing crop in alternate years) mango varieties. Despite plenty of polyembryonic genotypes available, no systematic effort has been made to observe their effects on regular and alternate-bearing varieties of mango with respect to alteration in physiochemical traits and molecular aspects. Hence the present study was undertaken with the objective to ascertain the role of rootstocks in regulating bearing in mango by using contrasting scion varieties on different rootstocks.

## Materials and methods

### Plant materials

The current study was conducted on 16 years old mango trees. For the first study, experimental material consisted of two contrasting mango varieties viz., alternate bearer ‘Dashehari’ and regular bearer ‘Amrapali’ grafted on two polyembryonic rootstocks namely ‘Olour’ and ‘Kurukkan’ and one commercially adapted monoembryonic rootstock i.e., non-descriptive seedlings (NDS). However, in the second study related to carbohydrate metabolism, experimental material contained four regular bearers (Pusa Arunima, Amrapali, Mallika, and Pusa Surya) mango varieties grafted on above mentioned three rootstocks. These trees were planted in a square system at 4m x4m spacing in 2006. The experimental orchard has typically described as subtropical semi-arid with hot and dry summer (May-June) followed by a cool winter (December to January). Our experimental location is situated under trans-Gangetic plains of agro-climatic zones of India. The soil of the experimental orchard was sandy loam with organic carbon 0.42%, pH 7.50, and EC (1:2) 0.14. During the course of the investigation, blocks were maintained as per the recommended cultural practices.

### Biochemical parameters

For all biochemical traits, fresh leaf samples and reproductive buds (S1 Fig) were collected one month before flower bud differentiation and at the time of fruit bud differentiation, respectively.

### Starch content in leaves and reproductive buds

Starch in leaf and bud samples was estimated using an anthrone reagent [20]. For the standard curve, a stock solution of standard glucose solution using 90 mg of glucose in 500 ml of distilled water to form a glucose concentration of 1 μ mole/1ml was prepared. After that working solution was prepared using 0.25, 0.5, 1.0, 1.5, and 2.0 ml of the stock standard solution into a series of test tubes and made up the volume to 2.5 ml with distilled water. Added 10 ml of freshly prepared Anthrone solution (100 mg of anthrone in 100 ml of chilled concentrated H _2_ SO_4_) and mixed well and heated the solution at 100°C in a boiling water bath for 15 minutes and cooled it immediately under tap water. Subsequently the absorbance was read at 620 nm in UV visible spectrophotometer (UV PLUS, MOTRA SCIENTIFIC).

### Sugar content in leaves and reproductive buds

These parameters were recorded on both leaf and reproductive buds. Sugars (total, reducing, and non-reducing) were estimated using the Nelson-Somogyi protocol [21]. Total sugars and reducing sugars were determined spectrophotometrically according to Hansen and Moller, [22] and Somogyi [21], respectively. Stock standard glucose solution: 45 mg of glucose in 500 ml of distilled water prepared to form a glucose concentration of 1 μ mole/2ml. Added 1 ml of Somogyi copper reagent and boiled it in a water bath for 12 minutes in a working solution of stock solution. Added 1 ml of arsenomolybdate reagent and made up the final volume to 10 ml. Subsequently, the absorbance was read at 530 nm in UV visible spectrophotometer (UV PLUS, MOTRAS SCIENTIFIC). Non-reducing sugars both in leaf and bud samples were calculated by subtracting the reducing sugars from the total sugar content.

### Protein content and C/N ratio in leaves and reproductive buds

Total soluble protein content was determined by the Lowry method [23]. The stock standard protein solution was prepared using 0.05 g of bovine serum albumin/50 ml of distilled water. A working standard solution was prepared by diluting 10 ml of the stock solution to 50 ml with water to obtain 200 μg protein/ml. Pipette out 0.2, 0.4, 0.6, 0.8, and 1.0 ml of the working standard solution into a series of test tubes. Folin Ciocalteau reagent of 0.5 ml was added and mixed well and incubated in dark for 30 minutes. Read the absorbance at 660 nm in UV visible spectrophotometer (UV PLUS, MOTRAS SCIENTIFIC). C: N ratio was calculated using CHNS analyzer [EURO EA Elemental analyzer (model- EURO VECTOR) which is based on the Dumas principle of dynamic flash combustion of the sample] [24]. For C: N ratio estimation, leaf and bud samples are crushed to powder with a pestle- mortar and the fine powder form of the sample was used for the analysis of C and N through a CHNS analyzer in percentage.

### Primary, secondary, and micronutrient estimation in leaf and bud tissues

Fresh leaf samples and flower buds were collected one month before flower bud differentiation and at the time of fruit bud differentiation, respectively. For this, terminal shoots were tagged in July, and leaves were harvested after 4 months of growth for recording biochemical traits and nutrient acquisition, while buds were collected in December. A total of 30 leaves /samples (3^rd^ and 4^th^ mature leaves from the top of the terminal shoot) were collected for sampling for nutrient observations. For estimation of P, K, Na, Ca, Mg, S, and micronutrients B, Fe, Mn, Cu, and Zn, one gram of the plant sample was digested in 4:1 nitric acid and perchloric acid (HNO_3_: HClO_4_) mixture. Phosphorus (%) (Vanadomolybdophosphoric yellow color method) [25] with spectrophotometer (Systronics UV- VS Spectrophotometer 117), Potassium (%) and Sodium with Flame photometer (Systronics Flame Photometer 128) [25] and Sulphur (%) (Turbidimetric method) [26] with spectrophotometer (Systronics UV- VS Spectrophotometer 117) estimated. Calcium (%), Magnesium (%) [25], and micronutrients- Fe, Mn, Cu, Zn (ppm) [27] with Motras Scientific Atomic Absorption Spectrophotometer recorded. Boron (ppm) estimated with Azomethine-H [28] with spectrophotometer- [Systronics UV- VS Spectrophotometer 117].

### Leaf gas exchange

Gas exchange parameters were recorded during 09:30–11:30 h in fully expanded mature leaves from each genotype under ambient light and CO_2_ level. Photosynthetic rate (*A*) μmole CO_2_ m^-2^ sec^-1^, transpiration rate (*E*) m. mole H_2_O m^-2^ sec^-1^, stomatal conductance (*g*_*s*_) m. mole H _2_ O m^-2^sec^-1^, and intercellular CO_2_ concentration (*Ci*) μmole CO_2_ m^-2^ sec^-1^ were measured using portable photosynthesis system (LCi-SD, ADC Bio Scientific Limited). Instantaneous water use efficiency (WUEi) was calculated by taking the ratio of photosynthetic rate and transpiration rate (*A/E*) and carboxylation capacity (CE) was calculated by taking the ratio of photosynthetic rate and internal CO_2_ concentration of the leaf (*A/Ci*). Observations on the leaf gas exchange were recorded under the following conditions: day temperature, 42.0°C; relative humidity (RH), 70% and sunshine hour, 8.7.

### Stomatal density

Stomata characteristics were measured by microscope (OLYMPUS CX33 and MagVision software). The distribution of stomata was analyzed using epidermal imprints of the lower surfaces of matured leaves following the method suggested by Sampson [29]. The stomata pore area index was calculated by multiplying pore area and stomata density. Chlorophyll fractions and carotenoid content were measured on mature leaves.

### Pigments

Chlorophyll (‘a’, ‘b’, total) and carotenoids were estimated [30]. A fully open mature leaf was taken as the experimental sample for chlorophyll estimation. Leaf samples of 50 mg were taken. Then it was cut into small pieces and put into a test tube. After that 10 ml of dimethyl sulphoxide (DMSO) was added to it and kept the solution in an oven at 60–62°C for 4 hours. Next-day readings were taken at 480 nm, 649 nm, and 665 nm, respectively through UV visible spectrophotometer (UV PLUS, MOTRAS SCIENTIFIC). Chlorophyll ‘a’, ‘b’, and total carotenoid content were estimated according to the formula given by Wellburn [31].

The formulas are given below:

Chlorophyll ‘a’ = (12.19 x A 665)- (3.45 x A 649) μg/ml

Chlorophyll ‘b’ = (21.99 x A 649)- (5.32 x A665) μg/ml

Total chlorophyll = Chl‘a’+ Chl‘b’

Total Carotenoids = [1000 x A 480 –(2.14 x Chl a) -(70.16 x Chl b)/220 μg/ml

### Carbohydrate metabolism-specific marker generation and validation

Four gene sequences viz., *Trehalose phosphate synthase*, *Sucrose phosphate synthase*, *Citrate synthase*, and *Alcohol dehydrogenase* of *Mangifera indica* L. were retrieved (S1 Table) (NCBI, www.ncbi.nlm.nih.gov). A nonredundant dataset (EG assembler web server, was generated [32]. Repetitive elements and vector sequences were removed. Only expressed sequence tags sequences were retained. Further, CAP3 [33] assembled the sequences into contigs with 80 percent overlap. SSRIT (simple sequence repeat identification tool; www.gramene.org/db/searches/SSRtool) was used to search for SSRs [34]. The SSR motifs generated by SSRIT were counted and the number of repetitions and frequency distribution was calculated. Primer 3 software (www.frodo.wi/mit.edu/primer3) was used to design primers. The parameters for designing primers were as follows: primer length between 21– 24bp, GC content between 25–62%, and optimum primer Tm between 55°C– 65°C. Thirty primers were synthesized for validation in 15 scion/rootstock combinations of mango. Carbohydrate metabolism genes namely *Trehalose phosphate synthase*, *Sucrose phosphate synthase*, *Citrate synthase*, and *Alcohol dehydrogenase* generated 9, 10, 5, and 6 primers, respectively (S1 Table). Fresh leaf samples were collected from scion/rootstock combinations [4 regular bearer (Amrapali, Pusa Arunima, Pusa Surya, and Mallika) and 1 alternate bearer (Dashehari)] and 03 Rootstocks i.e., Olour, Kurukkan (polyembryonic) and non-descriptive seedling (monoembryonic). Genomic DNA was extracted by the cetyltrimethylammonium ammonium bromide (CTAB) method with some modifications [35]. The genomic DNA was purified by successive RNase treatment. The quality of the extracted DNA was assessed by agarose gel electrophoresis and quantity by using Nanodrop 8000 spectrophotometer (Thermo Scientific, USA).

A total of 30 SSR loci were synthesized (S2 Table) for wet lab validation in 15 mango genotypes. The PCR was carried out in 20 μl reaction mixture containing 1 μl each primer (forward and reverse), 5μl of 25 ng/μl genomic DNA as a template, and 10μl of 2X PCR readymaster mix buffer (G Bioscience, USA). The volume was made up to 20μl with sterile distilledwater. To allow proper settling of the reaction mixture, PCR tubes containing the above components were capped and centrifuged at 5000 rpm for 1 minute. Amplification was carried out in a PE-Thermo cycler (C1000 Touch Thermal cycler, Bio-Rad, USA). Initial denaturation was carried out at 94°C for 5 minutes followed by 35 cycles of denaturation at 94°C, annealing at 55°C and extension at 72°C for 1 minute. The final extension was carried out at 72°C for 10 minutes and programmed to store at 4°C till the samples were processed further. PCR-amplified products were resolved in 3% high-resolution agarose gels. Electrophoresis was carried out at 100V for 3 to 4 hours. DNA profiles were visualized on a UV trans-illuminator and photographed on a gel documentation system (Alpha Innotech, USA). Power Marker 3.5 is used to calculate genetic distances between all-selected mango genotypes [36].

### Statistical analysis

The experiment was laid out in a Randomized Block Design (RBD) with a factorial arrangement. There were five replications with one tree in each replication. The data were subjected to statistical analysis of variance (ANOVA) using SAS 9.3 version software (SAS, USA INC) and significant differences were compared followed by DMRT at p≤ 0.05. The analysis of data was used to interpret the results and draw valid conclusions. The ClustVis web tool used for Heatmap generation [37] and for PCA analysis R/ADEGENET package was used [38].

## Results

### Starch content

#### Leaf tissues

Observation of starch content clearly showed significant variations by rootstock and scion individually as well as jointly (Table 1). Excluding the effect of variety, the mean effect of rootstock exhibited the highest leaf starch content (5.55 mg/g) in trees grafted on Kurukkan. However, trees raised on monoembryonic non-descriptive seedlings (NDS) showed to be a poor performer with respect to leaf starch content (3.73 mg/g). Irrespective of rootstock, the mean effect of the scion demonstrated the highest leaf starch content in Amrapali (4.59 g/g). The combined effect of rootstock and scion showed higher starch content when both varieties were grafted on Kurukkan, however, the trees of Dashehari exhibited the lowest leaf starch content while growing on NDS (monoembryonic). Interestingly polyembryonic rootstock Olour and Kurukkan produced similar leaf starch content in both varieties.

**Table 1:** Biochemical traits in leaf and reproductive bud tissues of regular and alternate bearer mango scion varieties as affected by rootstocks.

Scion/ rootstock	Leaf			Reproductive bud		
	Starch content (mg/g)						
	Dashehari	Amrapali	Mean	Dashehari	Amrapali	Mean
Olour	3.82^c^	3.96^c^	3.89^B^	1.97^e^	2.84^c^	2.41^C^
Kurukkan	5.62^a^	5.49^a^	5.55^A^	2.95^b^	2.83^c^	2.89^A^
NDS	3.14^d^	4.31^b^	3.73^C^	3.08^a^	2.54^d^	2.81^B^
Mean	4.19^B^	4.59^A^		2.67^B^	2.74^A^	
LSD (p≤0.05)						
Rootstock (R)	0.12			0.08		
Scion(S)	0.09			0.06		
RxS	0.16			0.11		
Scion/ rootstock	Total sugar (mg/g)					
	Dashehari	Amrapali	Mean	Dashehari	Amrapali	Mean
Olour	53.08^f^	70.81^c^	61.95^C^	57.52^d^	60.59^c^	59.05^C^
Kurukkan	74.85^b^	56.70^e^	65.78^B^	68.21^b^	71.85^a^	70.03^A^
NDS	88.76^a^	65.91^d^	77.33^A^	58.75^d^	72.04^a^	65.40^B^
Mean	72.23^A^	64.48^B^		61.49^B^	68.16^A^	
LSD (p≤0.05)						
Rootstock (R)	1.12			1.02		
Scion(S)	0.91			0.84		
RxS	1.58			1.45		
Scion/ rootstock	Reducing sugar (mg/g)					
	Dashehari	Amrapali	Mean	Dashehari	Amrapali	Mean
Olour	21.60^c^	43.56^a^	32.58^A^	48.32^a^	44.38^b^	46.35^A^
Kurukkan	42.85^a^	23.65^b^	33.25^A^	42.08^c^	43.74^cb^	42.91^C^
NDS	19.53^d^	19.66^d^	19.60^B^	42.67^cb^	46.61^a^	44.64^B^
Mean	27.99^B^	28.96^A^		44.35^A^	44.91^A^	
LSD (p≤0.05)						
Rootstock (R)	1.03			1.36		
Scion (S)	0.84			NS		
RxS	1.45			1.92		
Scion/ rootstock	Non reducing sugar (mg/g)					
	Dashehari	Amrapali	Mean	Dashehari	Amrapali	Mean
Olour	31.48^c^	27.25^d^	29.36^C^	9.20^d^	16.21^c^	12.71^C^
Kurukkan	32.01^c^	33.05^c^	32.53^B^	26.13^ba^	28.11^a^	27.12^A^
NDS	69.23^a^	46.25^b^	57.73^A^	16.08^c^	25.44^b^	20.76^B^
Mean	44.24^A^	35.52^B^		17.14^B^	23.25^A^	
LSD (p≤0.05)						
Rootstock (R)	1.44			1.71		
Scion (S)	1.17			1.40		
RxS	2.04			2.42		

*Mean of five replicates. Values with the same lower and upper letters are not significantly different at p<0.05, within the column, and in row, respectively.

### Reproductive bud tissues

Starch content of reproductive bud tissues was influenced significantly by rootstock, scion, and their interactions (Table 1). Regardless of the scion, the highest starch content was measured in bud tissues of trees grafted on Kurukkan rootstock and the lowest was observed in bud tissues of trees raised on Olour rootstock. The mean effect of scion showed that bud tissues of Amrapali had higher starch content. Furthermore, the combined effect of rootstock and scion exhibited the highest starch content in the Dashehari-NDS combination (3.08 mg/g) followed by the Dashehari-Kurukkan combination. It is worth mentioning that rootstock failed to influence the starch content in the buds of Amrapali.

### Sugars content

#### Leaf tissues

The total sugar, reducing sugar and non-reducing sugar content in leaf tissues showed a highly significant effect on rootstock, the scion (variety), and their interactions (Table 2). Without considering the effect of the scion, higher leaf total sugar and non-reducing sugar content were found in the leaves of trees grafted on NDS (77.33 mg/g), however, trees on Olour showed to be a poor performer with respect to leaf non reducing sugars content (29.36 mg/g). Further trees grafted on Kurukkan rootstock produced the highest leaf-reducing sugar (33.25 mg/g), which was similar statistically to trees grown on Olour rootstock (32.58 mg/g) but it was found the lowest when trees were grown on Olour (61.95 mg/g). Considering the effect of variety alone, Dashehari recorded the highest leaf total sugar content (72.23 mg/g) as well as nonreducing sugar, while it was Amrapali showed the highest reducing sugar (28.96 mg/g). The interaction effect between rootstock and scion clearly showed the highest leaf total sugar content in Dashehari-NDS (88.76 mg/g) followed by Dashehari-Kurukkan (74.85 mg/g) and Amrapali- Olour combinations (70.81 mg/g). Furthermore, a higher reducing sugar was noted in Amrapali- Olour (43.56 mg/g) and Dashehari-Kurukkan combinations (42.85 mg/g). Notwithstanding, the higher nonreducing sugar content was recorded in the Dashehari-NDS combination (69.23 mg/g). The lowest leaf-reducing sugar content in both varieties was observed on NDS (19.66 mg/g). However, Amrapali trees grafted on Olour exhibited the lowest nonreducing sugars content (27.25 mg/g). However, the lowest total sugar content was recorded in leaves taken from the trees of the Dashehari-Olour combination (53.08 mg/g).

**Table 2:** Primary and secondary nutrients in leaf and reproductive tissues of regular and alternate bearer scion varieties of mango as affected by rootstocks.

Scion/ rootstock	Leaf			Reproductive bud		
	N (%)					
	Dashehari	Amrapali	Mean	Dashehari	Amrapali	Mean
Olour	3.29^b^	1.71^d^	2.50^B^	1.52^c^	1.22^d^	1.37^C^
Kurukkan	1.69^d^	1.65^d^	1.67^C^	1.24^d^	1.73^b^	1.49^B^
NDS	3.53^a^	2.74^c^	3.14^A^	1.81^ba^	1.85^a^	1.83^A^
Mean	2.84^A^	2.04^B^		1.53^B^	1.60^A^	
LSD (p≤0.05)						
Rootstock (R)	0.09			0.07		
Scion(S)	0.07			0.05		
RxS	0.13			0.09		
Scion/ rootstock	P (%)					
	Dashehari	Amrapali	Mean	Dashehari	Amrapali	Mean
Olour	0.20^cb^	0.24^a^	0.22^A^	0.33^c^	0.33^c^	0.33^B^
Kurukkan	0.19^c^	0.20^b^	0.20^B^	0.36^b^	0.35^cb^	0.35^A^
NDS	0.19^d^	0.17^e^	0.18^C^	0.27^d^	0.40^a^	0.33^B^
Mean	0.19^B^	0.20^A^		0.32^B^	0.36^A^	
LSD (p≤0.05)						
Rootstock (R)	0.005			0.01		
Scion(S)	0.004			0.01		
RxS	0.007			0.02		
Scion/ rootstock	K (%)					
	Dashehari	Amrapali	Mean	Dashehari	Amrapali	Mean
Olour	0.37^d^	0.59^a^	0.48^A^	1.34^a^	0.92^e^	1.13^B^
Kurukkan	0.38^d^	0.50^c^	0.44^B^	1.19^cb^	1.24^b^	1.21^A^
NDS	0.55^b^	0.28^e^	0.41^C^	1.05^d^	1.13^cd^	1.09^B^
Mean	0.43^B^	0.46^A^		1.19^A^	1.09^B^	
LSD (p≤0.05)						
Rootstock (R)	0.01			0.06		
Scion (S)	0.01			0.05		
RxS	0.02			0.08		
Scion/ rootstock	Ca (%)					
	Dashehari	Amrapali	Mean	Dashehari	Amrapali	Mean
Olour	3.66^a^	1.44^d^	2.55^B^	4.00^c^	3.04^d^	3.52^C^
Kurukkan	1.41^d^	2.16^c^	1.79^C^	3.82^c^	4.29^b^	4.06^A^
NDS	3.39^b^	3.68^a^	3.54^A^	4.54^a^	3.10^d^	3.82^B^
Mean	2.82^A^	2.43^B^		4.12^A^	3.48^B^	
LSD (p≤0.05)						
Rootstock (R)	0.05			0.15		
Scion (S)	0.04			0.12		
RxS	0.07			0.21		

*Mean of five replicates. *Values with the same lower and upper letters are not significantly different at p<0.05, within the column, and in row, respectively.

### Reproductive bud tissues

Rootstock and scion individually as well as mutually impacted total sugar content and nonreducing sugars in bud tissues of scion significantly, scion failed to impact reducing sugar content in buds (Table 2). Excluding the effect of the scion, the highest total sugar and nonreducing sugar content in trees grafted on Kurukkan rootstock and lowest in buds taken from the trees raised on Olour rootstock, while contrasting results were obtained for reducing sugar. Irrespective of rootstocks a higher total sugar and nonreducing sugar content was found in the buds of Amrapali. The combined effect of rootstock and scion revealed higher total sugars content in the buds of the Amrapali- NDS combination (72.04 mg/g) or Amrapali-Kurukkan combination followed by Dashehari-Kurukkan, however, Dashehari-NDS (58.75 mg/g) and Dashehari-Olour (57.52 mg/g) combinations exhibited lower total sugars content in their buds. However, the Dashehari-Olour combination had the highest reducing sugar (48.32 mg/g), but it was statistically similar to Amrapali-NDS (46.61 mg/g) combinations. However lowest reducing sugar contents were recorded in Dashehari -Kurukkan combination (42.08 mg/g). The interaction between rootstock and scion exhibited the highest nonreducing sugars in Amrapali grafted on Kurukkan (28.11 mg/g) which showed statistical parity with Dashehari trees grafted on Kurukkan. However, the lowest non-reducing sugar content was recorded in buds of the Dashehari—Olour combination (9.20 mg/g).

### Protein content and C/N ratio

#### Leaf tissues

**Fig 1:**
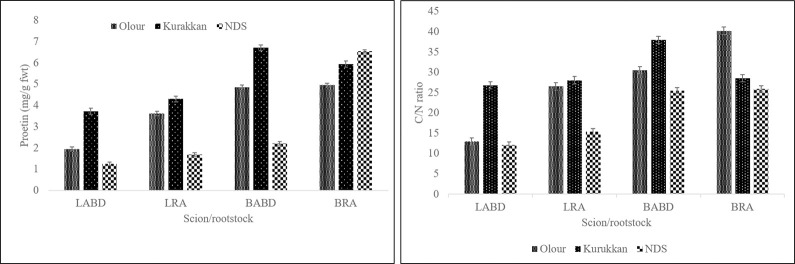
Protein content (A) and C/N ratio (B) of leaf and the reproductive bud of the regular bearer and alternate bearer mango varieties as affected by rootstocks. The LSD (p≤ 0.05) for protein; rootstock, 0.05, 0.14; scion variety, 0.05, 0.11; and rootstock x scion variety, 0.08, 0.20 for leaf and bud respectively and for C/N ratio; rootstock, 0.28, 1.43; scion variety, 0.23; NS, and rootstock x scion variety, 0.40, 2.02 for leaf and bud respectively. LABD; leaf of alternate bearer Dashehari, LRA, the Leaf of regular bearer ‘Amrapali’; BABD, the bud of alternate bearer ‘Dashehari’; BRA, the bud of regular bearer ‘Amrapali’.

Protein content and C/N ratio showed a highly significant effect of rootstock, and scion individually and mutually (p 0.001). Considering the effect of rootstock alone, both protein and C/N ratio as found to be higher when samples were taken from trees grafted on Kurukkan. Keeping in the mind effect of the variety only, Amrapali recorded the highest protein content (3.21 mg/g) and C/N ratio (23.31). Likewise, the effect of rootstock and scion jointly showed the highest leaf protein content and C/N ratio in Amrapali-Kurukkan rootstock followed by trees of Dashehari-Kurukkan and Amrapali-Olour combinations. However, the Dashehari-NDS combination exhibited the lowest protein and C/N ratio (1.25 mg/g) (Fig 1A and 1B).

#### Reproductive bud tissues

Rootstock and scion individually as well mutually affected protein content in buds significantly, while scion was unable to modify the C/N ratio in bud tissues (Fig 1A and 1B). Regardless of the scion, the peak protein content was found in trees grafted on Kurukkan, whereas, trees on Olour rootstock exhibited the highest C/N ratio. The Protein content and C/N ratio were lowest on NDS rootstock. The mean effect of the scion established higher protein content in Amrapali. Notwithstanding, mutually rootstock and scion showed the greatest protein content in Dashehari- Kurukkan combination (6.71 mg/g), which was statistically similar to Amrapali-NDS combination (6.54 mg/g). However, the Dashehari-NDS combination exhibited the lowest protein content in buds (2.21 mg/g). Furthermore, the highest C/N ratio was recorded in bud tissues of Amrapali-Olour (40.18) followed by Dashehari-Kurukkan (37.94) combinations. However, the lowest C/N ratio content was recorded in Dashehari- NDS combination (25.50), which was similar statistically to Amrapali-NDS (25.92). It is interesting to mention that NDS rootstock produced the lowest C/N ratio for both scions.

### Primary nutrients (N, P, and K)

#### Leaf tissues

Significant deviations were noticed for leaf N, and P and K content due to rootstock and scion individually as well as jointly (Table 2). The mean effect of rootstock alone clearly indicated that the highest leaf N content was found in trees grafted on NDS, while the highest leaf P content and K content was recorded in the trees grafted on Olour. Trees grafted on NDS found to be a poor accumulator of P and K, while it was Kurukkan on which trees exhibited the lowest leaf N content. Irrespective of rootstock, the mean effect of the variety established the highest leaf N content in Dashehari (2.84%), while Amrapali showed higher P and K content. Furthermore, the combined effect of rootstock and scion revealed significantly the highest leaf N content in Dashehari-NDS (3.53%) followed by Dashehari-Olour (3.29%) and Amrapali-NDS combinations (2.74%). On the other hand, the highest leaf P content was found in Amrapali- Olour (0.24%) followed by Amrapali-Kurukkan and Dashehari- Olour (0.20%). The highest leaf K in Amrapali- Olour combination (0.59%), was followed by Dashehari-NDS (0.55%) and Amrapali-Kurukkan (0.50%). Leaf N was found to be the lowest in leave tissues of Amrapali- Kurukkan (1.65%), which was similar statistically with Amrapali-Olour (1.71%) and Dashehari- Kurukkan (1.69%) combinations, however, the lowest leaf P and K content were recorded in the trees of Amrapali-NDS.

#### Reproductive bud tissues

The bud N, P, and K content were influenced significantly by rootstock, and scion individually and mutually (Table 2). The highest N content was measured in trees grafted on NDS rootstock, while it was Kurukkan produced the highest P and K content in the buds of the scion variety. The lowest N was found on Olour rootstock (1.37%), while Olour and NDS behave equally for bud P and K content. Excluding the effect of rootstock, higher N (4.57%) was measured in Amrapali, while Dashehari exhibited higher K content in its buds. The interaction of rootstock and scion exhibited the highest N in Amrapali-NDS (1.85%) followed by bud tissues of Amrapali-Kurukkan (1.73%). However, the lowest bud nitrogen content in the Amrapali-Olour (1.22%) and it was statistically similar to the Dashehari-Kurukkan (1.24%). It is important to mention that NDS rootstock produced the highest N in buds of both the scions. Furthermore, A higher P content was noted in Amrapali-NDS (0.40%) followed by Dashehari-Kurukkan (0.36%) which was non-significant with the Amrapali-Kurukkan combination. The lowest bud P content was observed in the Dashehari-NDS combination (0.27%). Notwithstanding, the Dashehari-Olour combination had the highest K content in bud tissues (1.34%) followed Amrapali-Kurukkan combination (1.24%). However, the lowest K content in buds was recorded in Amrapali- Olour combination (0.92%). It is interesting to mention that NDS rootstock showed the lowest K content in the buds of both scions.

### Secondary nutrients (Ca, Mg and S)

#### Leaf tissues

**Fig 2:**
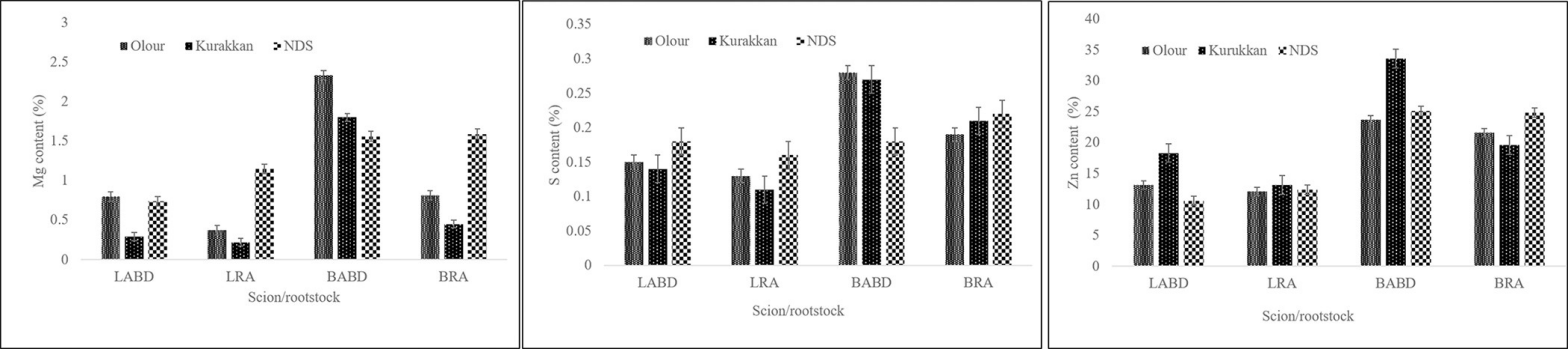
Content of Mg (A), S (B), and Zn (C) in leaf and reproductive bud tissues of the regular bearer and alternate bearer mango varieties as affected by rootstocks. The LSD (p≤ 0.05) for Mg; rootstock, 0.03, 0.08; scion variety, NS, 0.07; and rootstock x scion variety, 0.05, 0.12 for leaf and bud respectively and for S; rootstock, 0.009, 0.03; scion variety, 0.007, 0.02; and rootstock x scion variety, 0.10, 0.04 for leaf and bud respectively, for Zn; rootstock, 0.88, 1.30; scion variety, 0.72, 1.06; and rootstock x scion variety, 1.25, 1.83 for leaf and bud, respectively. LABD; leaf of alternate bearer Dashehari, LRA, the Leaf of regular bearer ‘Amrapali’; BABD, the bud of alternate bearer ‘Dashehari’; BRA, the bud of regular bearer ‘Amrapali’.

Leaf Ca and S content was influenced significantly by rootstock and scion alone and jointly, while variety failed to influence Mg content in leaf (Table 2, Fig 2A and 2B). The mean effect of rootstock exhibited that trees on NDS rootstock had the highest leaf Ca, Mg, and S content, however, significantly the lowest of all these nutrients in leaf tissues was observed in the trees on Kurukkan. Irrespective of rootstocks, Dashehari had higher leaf Ca and S content, while it was Amrapali that accumulated lower Mg content. The combined effect of rootstock and variety showed that Amrapali- NDS combination had the highest leaf Ca content (3.68%) which was statistically similar to Dashehari-Olour combinations (3.66%), leaf Mg accumulation in leaves of Amrapali- NDS combination (1.15%) followed by trees of Dashehari-Olour (0.80%) and Dashehari- NDS (0.74%). The highest leaf S accumulation was found in Dashehari-NDS (0.18%) followed by trees of Amrapali-NDS (0.16%) which was statistically similar to trees of Dashehari- Olour (0.15%). Dashehari- Kurukkan and Amrapali- Olour for Ca and Amrapali- Kurukkan combination for Mg and S, was found poor accumulator.

#### Reproductive bud tissues

The bud Ca content was manipulated significantly by rootstock, and scion independently as well mutually (Table 2 and Fig 2A and 2B). Regardless of the scion, the highest bud Ca content was measured in trees grafted on Kurukkan rootstock (4.06%), while trees on Kurukkan and Olour showed higher bud Mg and S content. The trees on Olour (3.52%) rootstock exhibited the lowest. The mean effect of scion showed higher bud Ca, Mg, and S content in Dashehari. Notwithstanding, rootstock and scion mutually indicated that the Dashehari-NDS combination (4.54%) had the highest bud Ca content followed by Amrapali-Kurukkan (4.29%) and Dashehari-Olour, which was non-significant with Dashehari-Kurukkan combinations. However, the lowest calcium content was observed in bud tissues of Amrapali on Olour (3.04%) which was statistically similar to the bud tissues of the same scion grafted on NDS rootstock (3.10%). The combined effect of rootstock and scion showed higher Mg and S content in buds of the Dashehari-Olour (2.33%, 0.28%) followed by Dashehari-Kurukkan (1.80%). The Mg content in bud tissues of Dashehari and Amrapali scions on NDS rootstocks was similar (1.56%). However, trees of Amrapali exhibited the lowest bud Mg content while grown on Kurukkan (0.45%). It is worth mentioning that rootstock failed to put forth any modification in S content in buds of Amrapali.

### Micronutrients (B, Mn, Fe, Cu, and Zn)

#### Leaf tissues

All studied micronutrients showed significant differences due to rootstock and scion individually as well as jointly (Table 3 and Fig 2C). Excluding the effect of variety, the mean effect of rootstock exhibited the highest B, Mn content in trees grafted on NDS, while more Cu accumulation on Olour rootstock and Zn was found to be the highest on Kurukkan rootstock. However, poor accumulation of B and Mn in leaf tissues was observed in trees raised on Kurukkan rootstock, and Cu and Zn on NDS rootstock. Irrespective of rootstock, Amrapali did excel for most of the micronutrients except leaf B and Zn content. Nonetheless, the combined effect of rootstock and variety showed higher B content in Dashehari- NDS combination (76.44 ppm) followed by Dashehari-Olour (64.89 ppm) and Dashehari-Kurukkan combinations. Whereas the highest Mn content was recorded in Amrapali- NDS (94.71 ppm) followed by Dashehari- NDS (82.26 ppm) combinations. Moreover, the highest leaf Fe was noted in Amrapali- NDS (134.70 ppm) followed by trees of Dashehari-Olour and Amrapali-Kurukkan combinations. The highest leaf Cu content in Dashehari- Olour (13.81 ppm), which showed statistical parity with Amrapali- Kurukkan, the highest Zn content in leaves of Dashehari—Kurukkan (18.33 ppm) followed by Dashehari-Olour which was non-significant with the rest of combinations except Dashehari-NDS. Amrapali-Olour and Amrapali-Kurukkan were found to be a poor accumulator of B, while Dashehari- Kurukkan combination poorly performed for Mn content and Zn content, and Dashehari -NDS showed for Fe content.

**Table 3:** Micronutrients contents in leaf and reproductive bud tissues of regular and alternate bearer scion varieties of mango as affected by rootstocks.

Scion/ rootstock	Leaf			Reproductive bud		
	B (ppm)					
	Dashehari	Amrapali	Mean	Dashehari	Amrapali	Mean
Olour	64.89^b^	35.91^d^	50.40^B^	78.58^b^	60.44^c^	69.51^B^
Kurukkan	52.44^c^	34.67^d^	43.56^C^	43.02^e^	64.00^c^	53.51^C^
NDS	76.44^a^	54.20^c^	65.36^A^	56.13^d^	88.53^a^	72.33^A^
Mean	64.59^A^	41.59^B^		59.25^B^	70.99^A^	
LSD (p≤0.05)						
Rootstock (R)	0.06			2.66		
Scion(S)	0.05			2.17		
RxS	0.08			3.76		
Scion/ rootstock	Mn (ppm)					
	Dashehari	Amrapali	Mean	Dashehari	Amrapali	Mean
Olour	48.24^c^	44.01^d^	46.13^B^	44.10^bc^	42.12^c^	43.11^B^
Kurukkan	41.55^e^	46.80^c^	44.18^C^	45.90^b^	53.65^a^	49.77^A^
NDS	82.26^b^	94.71^a^	88.49^A^	55.86^a^	43.14^c^	49.50^A^
Mean	57.35^B^	61.84^A^		48.62^A^	46.30^B^	
LSD (p≤0.05)						
Rootstock (R)	0.15			1.74		
Scion(S)	0.12			1.42		
RxS	0.21			2.46		
Scion/ rootstock	Fe (%)					
	Dashehari	Amrapali	Mean	Dashehari	Amrapali	Mean
Olour	116.80^b^	86.64^e^	101.72^B^	173.12^a^	112.44^d^	142.78^A^
Kurukkan	70.94^f^	104.02^c^	87.48^C^	173.44^a^	89.88^e^	131.66^B^
NDS	94.74^d^	134.70^a^	114.72^A^	128.80^c^	155.32^b^	142.06^A^
Mean	94.16^B^	108.45^A^		158.45^A^	119.21^B^	
LSD (p≤0.05)						
Rootstock (R)	3.00			1.74		
Scion (S)	2.45			1.42		
RxS	4.24			2.46		
Scion/ rootstock	Cu (%)					
	Dashehari	Amrapali	Mean	Dashehari	Amrapali	Mean
Olour	13.81^a^	8.58^b^	11.20^A^	14.66^cb^	18.13^a^	16.40^A^
Kurukkan	8.21^cb^	13.30^a^	10.76^B^	16.27^b^	18.06^a^	17.17^A^
NDS	5.19^d^	7.65^c^	6.42^C^	14.25^c^	15.60^cb^	14.92^B^
Mean	9.07^B^	9.84^A^		15.06^B^	17.26^A^	
LSD (p≤0.05)						
Rootstock (R)	0.44			1.24		
Scion (S)	0.36			1.02		
RxS	0.62			1.76		

*Mean of five replicates. Values with the same lower and upper letters are not significantly different at p<0.05, within the column, and in row, respectively.

#### Reproductive bud tissues

Rootstock and scion independently and mutually affected bud B, Mn, Fe, Cu, and Zn content significantly (Table 3 and Fig 2C). Regardless of the scion, the highest B and Fe content was measured in trees grafted on NDS rootstock, but it was statistically similar with trees on Olour for later one. The trees grafted on the Kurukkan rootstock were found to be a very poor accumulator of B in buds, contrary to trees on this rootstock that showed higher Mn, Zn, and Cu content, but similar statistically with Olour for later one. The mean effect of scion showed a higher bud B and Cu content in Amrapali, while Dashehari exhibited higher Mn, Zn, and Fe content. Furthermore, rootstock and scion mutually exhibited the highest B in bud tissues of the Amrapali-NDS combination (88.53 ppm) followed by the Dashehari-Olour combination (78.58 ppm) and Amrapali-Kurukkan, which showed statistical similarity with Amrapali-Olour combinations. However, the lowest boron content was observed in the bud tissues of Dashehari on Kurukkan rootstock (43.02 ppm). Furthermore, the highest bud Mn content in the Dashehari-NDS combination (55.86 ppm), was statistically similar to the Amrapali-Kurukkan combination (53.65 ppm). However, Amrapali-Olour exhibited the lowest bud Mn content (42.12 ppm), which was statistically similar to the bud tissues of the same Amrapali- NDS combination (43.14 ppm). The highest Fe content in buds in Dashehari-Kurukkan (173.44 ppm) was statistically similar to the Dashehari-Olour rootstock (173.12 ppm). However, Amrapali—Kurukkan combination was found to be a poor accumulator of bud Fe (89.88 ppm). Amrapali had the highest Cu in buds while grown on either Olour (18.13 ppm) or Kurukkan (18.06 ppm) rootstocks. However, the lowest Cu content in bud tissues was observed in the Dashehari-NDS combination (14.25 ppm). Meanwhile, the highest bud Zn content was measured in Dashehari-Kurukkan (33.60 ppm), followed by Dashehari-NDS (25.14 ppm) which was statistically similar to the bud tissues of the Dashehari-Olour (23.74 ppm) and with the bud tissues of Amrapali-NDS (24.86 ppm). However, trees of Amrapali on Kurukkan exhibited the lowest Zn content (19.62 ppm).

### Physiological traits

#### Leaf gas exchange

Observations of leaf gas exchange traits exhibited a significant effect on rootstock, scion, and their joint effect on photosynthetic rate (A), transpiration rate (E), and Intercellular CO_2_ Concentration (Ci) but rootstock and scion failed to influence gas significantly (Table 4). Excluding the effect of the scion, the highest A and E were measured in trees grafted on Kurukkan rootstock, while the greatest concentration was noted in trees grafted on NDS followed by trees on Kurukkan. Irrespective of rootstocks the mean effect of the scion demonstrated a higher A in Amrapali (1.24-fold). The mean effect of the scion showed that the leaf tissues of Dashehari had the highest E (3.23 m mole H_2_O m ^-2^ sec ^-1^). The mean effect of the scion demonstrated a higher Ci concentration in Dashehari (4.51%) than in Amrapali. The combined effect of rootstock and scion showed a higher photosynthetic rate in Amrapali—Kurukkan combination (10.60 μmole CO_2_ m ^-2^ sec ^-1^) followed by Amrapali-Olour and Dashehari-Kurukkan combinations (9.57 μmole CO_2_ m ^-2^ sec ^-1^), while the highest gs was found in Amrapali-Kurukkan which was non-significant with Dashehari-NDS and Amrapali-Olour combinations. A higher E was recorded in Dashehari-Kurukkan (3.77 m mole H_2_O m ^-2^ sec ^-1^) followed by Amrapali-Kurukkan, which was similar statistically with Dashehari-NDS combinations. Furthermore, Dashehari-Olour (272.80 μmole CO_2_ m ^-2^ sec ^-1^) exhibited the highest Ci which was statistically similar to Amrapali—NDS combination (262.80 μmole CO_2_m ^-2^ sec ^-1^). It was found to be the lowest in Dashehari -Olour combination (6.07 μmole CO_2_m ^-2^ sec ^-1^). The lowest E was recorded in the Amrapali-NDS scion combination (2.09 m mole H_2_O m ^-2^ sec ^-1^), but the Amrapali- Olour combination (226.20 μmole CO_2_ m ^-2^ sec ^-1^) showed the lowest Ci.

**Table 4:** Leaf gas exchange parameters, intrinsic water use efficiency and carboxylation capacity of regular and alternate bearer scion varieties of mango as affected by rootstock.

Scion/ rootstock	Photosynthetic rate (μmole CO_2_ m^-2^ sec^-1^)			Stomatal conductance (mole H_2_O m^-2^ sec^-1^)		
	Dashehari	Amrapali	Mean	Dashehari	Amrapali	Mean
Olour	6.07^e^	9.57^b^	7.82^B^	0.09^b^	0.10^ba^	0.10^A^
Kurukkan	8.49^c^	10.60^a^	9.55^A^	0.09^b^	0.12^a^	0.11^A^
NDS	7.50^d^	7.33^d^	7.41^B^	0.12^a^	0.10^b^	0.11^A^
Mean	7.35^B^	9.17^A^		0.10^A^	0.11^A^	
LSD (p≤0.05)						
Rootstock (R)	0.53			NS		
Scion (S)	0.44			NS		
RxS	0.76			0.02		
Scion/ rootstock	Transpiration rate (m mole H_2_O m^-2^ sec^-1^)			Intercellular CO_2_concentration (μ mole CO_2_ m^-2^ sec^-1^)		
	Dashehari	Amrapali	Mean	Dashehari	Amrapali	Mean
Olour	2.68^c^	2.64^c^	2.66^B^	272.80^a^	226.20^c^	149.50^BA^
Kurukkan	3.77^a^	3.28^b^	3.52^A^	243.40^b^	240.40^cb^	241.90^B^
NDS	3.25^b^	2.09^d^	2.67^B^	246.00^b^	262.80^a^	254.40^A^
Mean	3.23^A^	2.67^B^		254.10^A^	243.13^B^	
LSD (p≤0.05)						
Rootstock (R)	0.14			10.94		
Scion (S)	0.11			8.93		
RxS	0.20			15.47		
Scion/ rootstock	Instantaneous water use efficiency			Carboxylation capacity		
	Dashehari	Amrapali	Mean	Dashehari	Amrapali	Mean
Olour	0.002^c^	0.004^a^	0.003^A^	0.02^c^	0.04^a^	0.03^B^
Kurukkan	0.002^c^	0.003^b^	0.003^A^	0.04^b^	0.04^a^	0.04^A^
NDS	0.002^c^	0.004^ba^	0.003^A^	0.03^b^	0.03^b^	0.03^B^
Mean	0.002^B^	0.004^A^		0.03^B^	0.04^A^	
LSD (p≤0.05)						
Rootstock (R)	NS			0.003		
Scion (S)	0.02			0.002		
RxS	0.03			0.004		

*Mean of five replicates. Values with the same lower and upper letters are not significantly different at p<0.05, within the column, and in row, respectively.

#### Instantaneous water use efficiency (WUEi)

Instantaneous water use efficiency (WUEi) was affected significantly by scion and interaction between rootstock and scion, however, rootstock failed to exert any effect on WUEi (Table 4). Considering the effect of scion alone, the mean effect displayed a higher value of WUEi in Amrapali (50.22%) than in Dashehari. The interaction effect of rootstock and scion witnessed the highest WUEi in Amrapali- Olour (3.64) combination followed by Amrapali-NDS, which showed a statistical party with Amrapali-Kurukkan combinations. It is interesting to mention that Dashehari exhibited lower WUEi on all rootstocks, even lower than the lowest value of WUEi on any combination of the rootstock with Amrapali.

#### Carboxylation capacity (CE)

Leaf carboxylation capacity (CE) was influenced significantly by rootstock, scion, and their interaction (Table 4). Regardless of the scion, the highest CE was measured in trees grafted on Kurukkan rootstock (0.04), and the carboxylation capacity in leaves of trees raised on Olour and NDS was similar (0.03). Regardless of rootstock, the mean effect of the scion showed that Amrapali had 33.33% higher CE than Dashehari. Mutually rootstock and scion also exhibited a highly significant variation with regard to CE and combination of Amrapali—Olour and Amrapali- Kurukkan, Dashehari-Kurukkan combinations (0.04). The lowest CE was observed in Dashehari scion while grown on Olour rootstock (0.02).

#### Total chlorophyll and carotenoids

**Fig 3:**
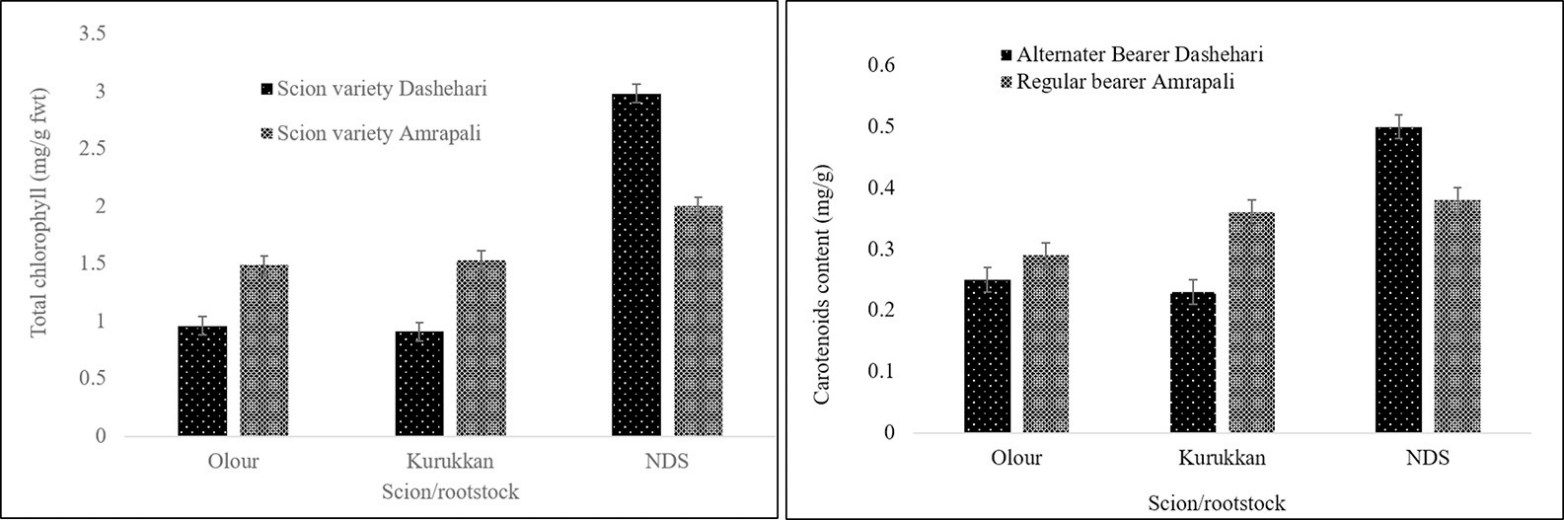
Total chlorophyll (A) and carotenoid content (B) of leaf tissues of the regular bearer and alternate bearer mango varieties as affected by rootstocks. The LSD (p≤ 0.05) for total chlorophyll; rootstock, 0.07; scion variety, 0. NS; and rootstock x scion variety, 0.10 and for total carotenoids; rootstock, 0.02; scion variety, NS; and rootstock x scion variety, 0.03. LABD; leaf of alternate bearer Dashehari, LRA, the Leaf of regular bearer ‘Amrapali’; BABD, the bud of alternate bearer ‘Dashehari’; BRA, the bud of regular bearer ‘Amrapali’.

Rootstock alone and jointly with scion affected total chlorophyll and carotenoid content significantly (Fig 3A and 3B), while scion alone failed to influence total chlorophyll content. The mean effect of rootstock exhibited the highest total chlorophyll and carotenoid content in trees grafted on NDS rootstock, whereas leaves of trees on Olour and Kurukkan rootstock shows statistically similar values. The combined effect of rootstock and scion showed higher total chlorophyll and carotenoids content in leaves of trees of Dashehari-NDS followed by leaves of trees of Amrapali on NDS rootstock and Amrapali on Kurukkan, which was non-significant with Amrapali on Olour rootstock. The trees of Dashehari either on Olour or Kurukkan rootstock exhibited lower values of total chlorophyll.

#### Stomatal density

**Fig 4:**
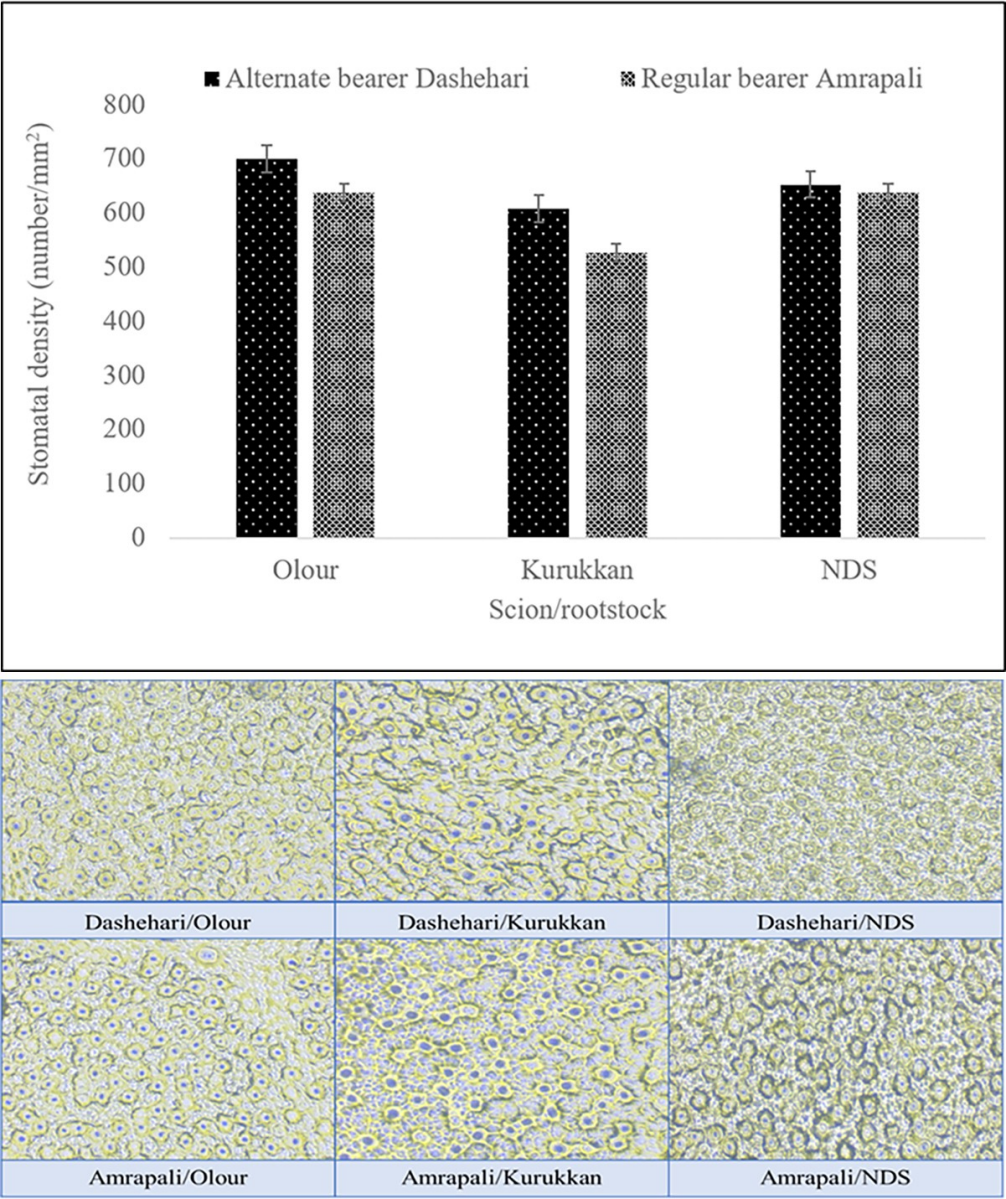
Stomatal density (A) and pictorial representation of stomatal density (10 X) (B). The LSD (p≤ 0.05) rootstock, 0.05; scion variety, 0.05; and rootstock x scion variety.

Leaf stomatal density was influenced significantly by rootstock, scion, and their interactions (Fig 4A and 4B). Regardless of the scion, the highest stomatal density was measured in trees grafted on Olour rootstock and lowest in trees grafted on Kurukkan rootstock. The mean effect of the scion showed that Dashehari had the highest stomatal density (1.10-fold) than Amrapali. The joint effect of rootstock and scion exhibited higher stomatal density in Dashehari-Olour (700.40) followed by Dashehari- NDS (652.60), which was statistically similar with trees of Amrapali -NDS (637.60) and Amrapali-Olour (637.20) combinations. However, Amrapali-Kurukkan (526.40) combination had the lowest stomatal density.

### Statistical analysis

To identify the key parameters or assess Heatmap and Principal Component analysis data were generated. Principal component analysis of leaf biplot of morphological and physiological traits of 6 mango genotypes under 33 treatments. The two physiological parameters (SD and Ci) allow the separation of 6 mango genotypes and the variation at PC1 (99.5%) and PC2 (0.2%) (S2 Fig). Whereas Principal Component analysis of bud biplot of morphological and physiological traits of 6 mango genotypes under 19 treatments (S3 Fig). The three physiological parameters (Fe, TS, and B) allow the separation of 6 mango genotypes the variation at PC1 (96.6%) and PC2 (2.3%) (Table 5).

**Table 5:** Variance explained by Principal Component (6 Basic Component) in scion/rootstock combinations of mango.

Leaf	PC1	PC2	PC3	PC4	PC5	PC6
Individual	0.9954	0.0021	0.0014	0.0005	0.0003	0.0001
Cumulative	0.9954	0.9975	0.9990	0.9995	0.9999	1
Bud	PC1	PC2	PC3	PC4	PC5	PC6
Individual	0.9657	0.0227	0.00687	0.00251	0.00206	0.001
Cumulative	0.9657	0.9884	0.9953	0.9978	0.9999	1

**Fig 5:**
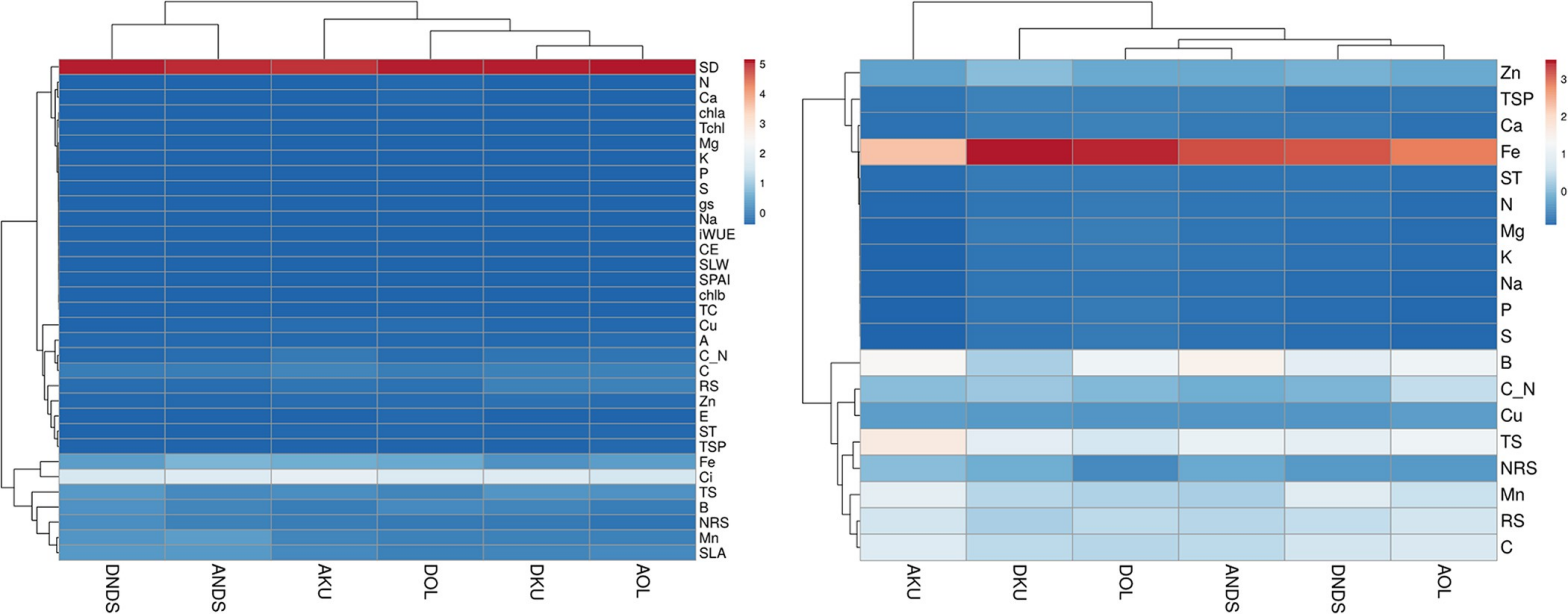
Heatmap and hierarchical clustering for physiological and nutritional parameters of the leaf (A) and bud tissues (B) in alternate and regular bearer mango varieties grafted on different rootstocks. A; Amrapali, D; Dashehari, Ol; Olour, Ku; Kurukkan, NDS; non-descriptive seedlings.

Heat map generated for leaf and bud tissues using biochemical and physiological parameters and clustering analysis of leaf parameters showed three main groups represented 33 traits (Fig 5A). Here, in the group, an SD is highly upregulated in groups b, C, and RS are upregulated. Ci and Fe were highly expressed in group c. Clustering analysis using bud parameters of scion/rootstock combinations showed two main groups were represents 19 traits. Here, in the group, Fe is highly upregulated followed by Zn. In group b, TS and B are upregulated followed by Mn, RS, and C (Fig 5B).

### Scion/Rootstock interactions via carbohydrate metabolism specific markers

**Fig 6:**
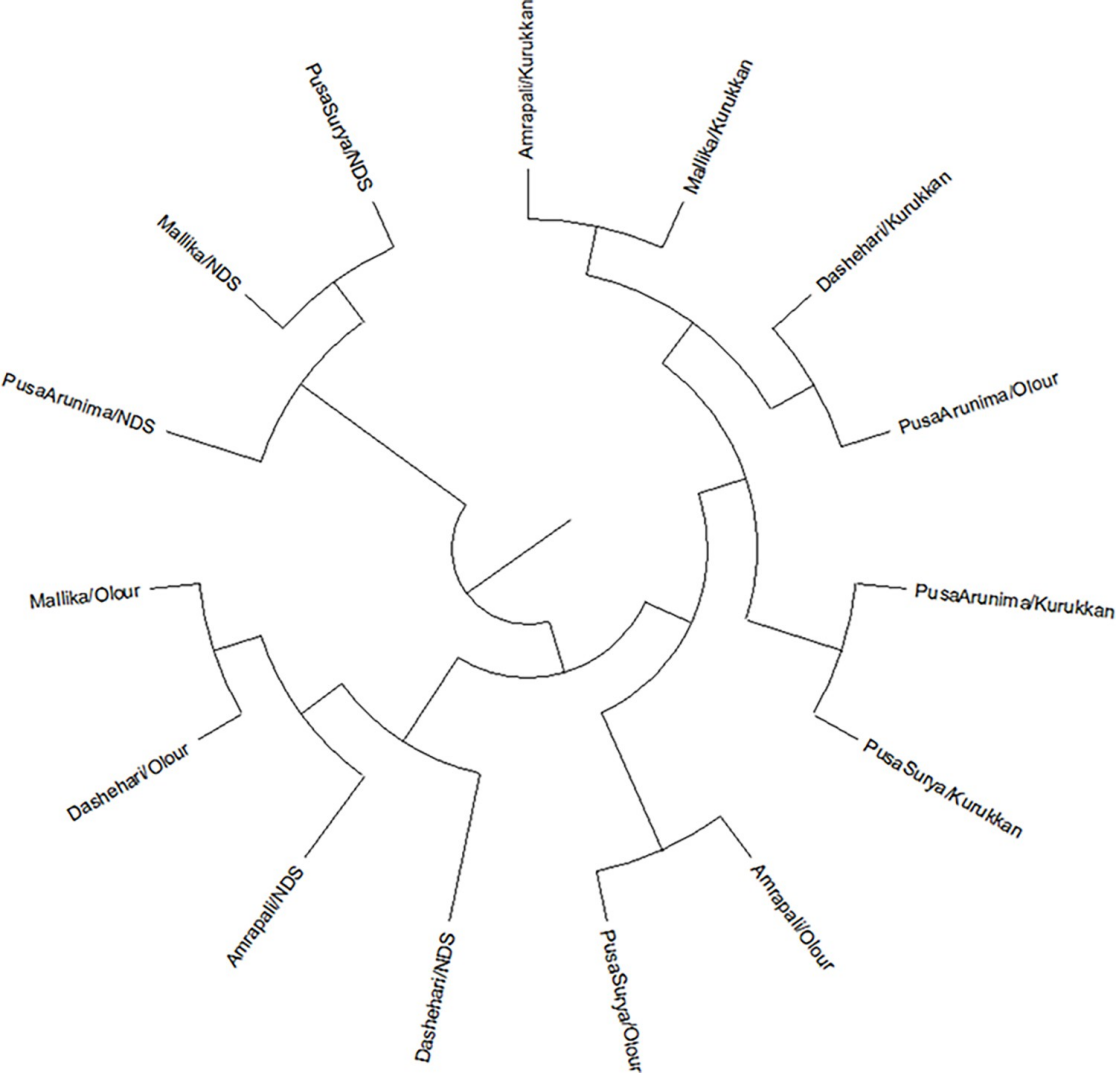
Genetic tree using carbohydrate metabolism specific primers of the regular bearer and alternate bearer mango varieties as affected by rootstocks.

DNA yield was found to vary in different scion/rootstock combinations and the highest yield in Dashehari/Olour (1363.30 ng/μl) while it was the lowest in Pusa Arunima/Olour (407.80 ng/μl) with 790.91 ng/μl average yield. The average value of DNA quality on the basis of nanodrop reading (A260/280) was 2.00 and the maximum value was found in Pusa Surya/Non- descriptiveseedling (2.20) and the minimum value was found in Dashehari/Kurukkan (1.82). Details of carbohydrate metabolism-specific genes used for primer designing are given in S1 and S2 Tables. A total of 33 alleles were amplified among carbohydrate metabolism-specific markers, which varied from 2 to 3 alleles with a mean of 2.53 per locus. The major allelic frequency (MAF) ranged from 0.40 to 0.87 among the carbohydrate metabolism-specific markers with a mean value of0.56 per locus. The carbohydrate metabolism-specific markers NMSPS2 had the highest allelic frequency (0.87), while NMTPS9 had the lowest value (0.40). Maximum and minimum PIC value was found for NMSPS10, NMTPS9 primers (0.58), and NMSPS2 (0.20), respectively (Table 6). However, the average PIC value was 0.44 per locus. The gene diversity of the carbohydrate metabolism-specific primers was calculated for 13 polymorphic primers, which ranged from 0.23 to 0.66 with an average of 0.52 per locus (Table 6). The SSR profile of NMAD1 in15 scion/ rootstock combinations of mango is shown in S4 Fig. A total of 4 carbohydrate metabolism-specific markers, viz., NMCS1, NMCS2, NMSPS10, and NMTPS9 had the PIC value>0.5 for the mango genotypes, which indicated their high discrimination power. Cluster analysis revealed that scion grafted on Kurukkan rootstock clustered together except for Pusa Arunima on Olour rootstock (Fig 6). For Amrapali/Kurukkan maximum dissimilar value was found with Dashehari/NDS (0.7097) and the minimum dissimilar value was found with Mallika/Kurukkan (0.0750). For Amrapali/Olour maximum dissimilar value was found with Amrapali/NDS (0.7357) and the minimum dissimilar value was found with Pusa Surya/Olour (0.2884). For Amrapali/NDS maximum dissimilar value was found with Amrapali/Olour (0.7357) and the minimum dissimilar value was found with Dashehari/Olour (0.3520). For Dashehari/Kurukkan maximum dissimilar value was found with Dashehari/Olour, Mallika/Olour (0.6290), and a minimum dissimilar value was found with Pusa Arunima/Olour, Mallika/Kurukkan (0.2135). For Dashehari/NDS maximum dissimilar value was found with Amrapali/Kurukkan, and Mallika/Kurukkan (0.7097), and a minimum dissimilar value was found with Pusa Surya/Olour (0.3577). For Dashehari/Olour the maximum dissimilar value was found with Amrapali/Kurukkan (0.6347) and the minimum dissimilar value was found with Mallika/Olour (0.2078).

**Table 6:** Genetic variability indices of the 13 polymorphic carbohydrate metabolism specific primers among the set of 15 scion/rootstock combinations of mango.

S.N.	Marker ID	Annealing temp. (Ta) (°C)	Allele size (bp)	Maf	An	GD	Ho	PIC
1	NMAD1	55	200–210	0.60	2	0.48	0.00	0.36
2	NMAD2	55	240–250	0.57	2	0.49	0.47	0.37
3	NMAD3	55	250–260	0.73	3	0.43	0.00	0.39
4	NMAD4	55	240–250	0.53	2	0.50	0.27	0.37
5	NMAD6	55	170–180	0.53	3	0.55	0.13	0.46
6	NMCS1	55	900–910	0.47	3	0.64	0.00	0.57
7	NMCS2	55	700–710	0.47	3	0.63	0.00	0.56
8	NMCS3	55	500–510	0.60	3	0.55	0.13	0.48
9	NMSPS2	55	190–210	0.87	2	0.23	0.27	0.20
10	NMSPS3	55	160–170	0.57	2	0.49	0.47	0.37
11	NMSPS10	55	200–210	0.43	3	0.65	0.20	0.58
12	NMTPS1	55	180–200	0.50	2	0.50	0.47	0.37
13	NMTPS9	55	180–200	0.40	3	0.66	0.00	0.58
Mean				0.56	2.53	0.52	0.18	0.44

Where: Maf = major allele frequency, An = Allele number, GD = gene diversity, Ho = observed heterozygosity, PIC = polymorphism information content

## Discussion

### Biochemical traits

In the present study, we found that both Olour and Kurukkan rootstocks promoted the accumulation of bud starch in Amrapali, while bud total sugar content was promoted maximum in variety Amrapali. Further, trees of alternate bearer ‘Dashehari’ were able to transport more sugar from leaves to buds while grown on Kurukkan rootstock. Kurukkan rootstock did excellent for higher transport of non-reducing sugars in the buds of both varieties. Earlier, [39] also reported that rootstocks significantly affect the soluble protein content of scion trees of citrus ‘Shatangju’ mandarin. Furthermore, the C/N ratio in scion trees of mango was significantly influenced by different rootstocks in our findings. Furthermore, Satisha et al. [40] have also found a significant effect of rootstocks on protein content in the leaves of the grapevine. Guitton et al., [41] stated that the struggle for carbohydrates between the developing fruit and close apical buds lead to local carbon reduction and decreased cellular activity in the vegetative meristems thus, blocking the onset of floral development.

### Nutrient content

Non descriptive seedlings (monoembryonic desi type, NDS) rootstock found to be the good accumulator of N and S in leaf tissues of both regular and alternate bearer scion varieties as well as Mg content in leaves of regular bearer ‘Amrapali’. Likewise, this rootstock also showed a higher increase of leaf K in ‘Dashehari’ and Ca in ‘Amrapali’. NDS rootstock was more able to transport N, P, Mg, Mn, and Fe in bud tissues of ‘Amrapali’, but transport Ca in buds of Dashehari. While B, Mn was retained in leaves of alternate bearer ‘Dashehari’. Notwithstanding, Kurukkan rootstock enhanced Zn content in the leaf tissue of Dashehari, but was able to transfer P, Mn, and S content in buds of Dashehari, K, and Ca content in buds of Amrapali. However, Kurukkan rootstock exhibited superiority to retain Fe and Cu in leaves of regular bearer ‘Amrapali’. On the otherhand, the rootstock Olour was able to retain more P in both the scion varieties and K in regular bearer ‘Amrapali’ and Ca and K and Fe contents in alternate ‘Dashehari’, however, Olour rootstock transported more B, Fe, and Cu in the buds of alternate bearer ‘Dashehari’. Overall, we could say that rootstock has differential capacity to absorb and translocate nutrients for various scion varieties. Differences in nutrient accumulation in scion varieties as a result of rootstock-scion combination may be due to various reasons such as the structure of the root system, variations in root CEC, characteristics of root exudates [42], due to the interspecific difference among rootstocks with respect to nutrient absorption and the transference of this trait to the scion variety [43] and phenotype of the scion cultivars [44]. Differences in K^+^ accumulation in leaf tissues of scion cultivars may be due to variation in the absorption capacity of rootstock or differences in the incorporation of K^+^ ions into the xylem and their translocation from root to shoot [45]. Earlier, differences in the accumulation and translocation of nutrients in different rootstock -scion varieties also reported [18, 46–49].

### Physiological parameters

Kurukkan rootstock expressed its dominance for higher photosynthetic rate (*A*) in alternate bearing ‘Dashehari’ and transpiration rate (*E*) and stomatal conductance (*g*_*s*_) in regular bearer ‘Amrapali’, whereas NDS rootstock encouraged stomatal conductance in ‘Dashehari’ and internal CO_2_ concentration (Ci) in Amrapali. Furthermore, our results suggested that Olour promoted internal CO_2_ concentrations in alternate bearer ‘Dashehari’ and intrinsic water use efficiency (WUEi) in regular bearer ‘Amrapali’. Based on our results, it could be argued that both Kurukkan and NDS rootstock, and Olour and Kurukkan were able to promote carboxylation capacity in Dashehari and Amrapali, respectively. Olour could modify stomatal density in alternate ‘Dashehari’, but we observed no change in stomatal density in regular bearer ‘Amrapali’. Earlier Zhou et al. [50] also reported variation in stomatal density in scion varieties of apple and found more stomatal density in dwarfing rootstocks M.9 and B.9 than in vigorous rootstock Baleng on apple scion variety ‘Red Fuji’. NDS rootstock promoted total chlorophyll in both irregular and regular bearer varieties and carotenoid content in irregular bearer ‘Dashehari’, however, in the regular variety ‘Amrapali’, NDS and Kurukkan can modify total carotenoids content. It has been shown that photosynthesis variables, including stomatal conductance, intercellular CO_2_ concentration, and transpiration, were all significantly influenced by the rootstock [51]. It was reported in previous study that rootstock genotype affected the leaf gas exchange parameters in different rootstock-scion combinations [18, 52].

### Genetic characterization of mango rootstocks and scion/rootstock combinations using carbohydrate metabolism-specific primers

Flowering regularity, time, and intensity are critical factors directly associated with the economic status of mango growers. Flowering is usually related to existing native environmental conditions, heredity, nutrition status, and hormonal aspects in mango [53]. Several molecular studies [54–56] indicated the involvement of carbohydrate metabolism-specific genes in regulating the flowering or bearing of fruit crops. Further, molecular studies reported the involvement of *Trehalose Phosphate Synthase* genes in the flowering of crops like avocados [56] and apples [57]. Han et al. [58, 59] observed the involvement of *Citrate Synthase* (CS) genes in the flowering of transgenic tobacco and transgenic *Arabidopsis* plants. Micallef et al. [60] reported the involvement of the *Sucrose Phosphate Synthase* (*SPS*) gene in the flowering of transgenic tomatoes. Studies by Gregerson et al. [61]; Eldik et al. [62] showed the involvement of *Alcohol dehydrogenase* gene activity in the flowering of potatoes.

Molecular markers like SSRs and SNPs are distributed throughout the genome. These markers have diverse applications in fruit crops. It could be well utilized for varietal identification, genetic diversity, gene tagging, pedigree analysis, and marker-assisted breeding. Rapid assay of molecular markers like SSR, ISSR, SCAR, RAPD, and AFLP can be done via PCR [63]. A molecular study for flowering in mango is limited due to the meager functional markers associated with flowering. Microsatellites or simple sequence repeats (SSR) as DNA markers are more advantageous over various other markers. These are more polymorphic, co-dominant, highly abundant, readily transferable, and analytically simple in nature. SSRs are found to be highly variable as compared to RAPD or RFLP and have been reported to be utilized extensively in various genomic studies. However, the genomic library-dependent approach for SSR marker development is time-consuming [64]. With the advances in bioinformatics, it is possible to mine and analyze large-scale EST datasets efficiently. Further, *in silico* database offers the opportunity to identify SSRs in ESTs through data mining. It provides a simple and cost-efficient approach for the development of SSR markers in plants. Microsatellite markers have been successfully used in mango breeding.

Identification of linked markers underlying major economic traits, such as regular bearing, fruit quality, yield-associated traits, and disease resistance or stress tolerance in mango is extremely essential in mango [65]. Azam et al. [66] reported that SSR markers have a significant association with economic traits like reducing sugars, total sugars, titratable acidity, ascorbic acid, the total number of flowers, and no. of hermaphrodite flowers in mango. Lal et al. [65] used genome-wide association mapping in 87 polymorphic genic-simple sequences repeats (SSR) markers, 17 important pomological features in mango mapped. Using the GLM, were able to identify 23 genic-SSR markers that were linked to 13 different pomological features. Moreover, the present study is an initiative in the direction of the development of functional carbohydrate metabolism-specific microsatellite markers in mango that could be efficiently used for genetic mapping studies of segregating mango populations. These newly designed markers are well utilized in 15 scion/ rootstock combinations of mango to characterize them for regular and alternate bearers based on their carbohydrate metabolism levels. If the location of this genetic material (marker) has been determined and the benefits of the marker have been identified, DNA from other rootstocks can be quickly screened for the gene of interest. In the present study out of the 30-carbohydrate metabolism-specific primers tested, 13 gave polymorphic amplicons, they are namely NMAD1, NMAD2, NMAD 3, NMAD4, NMAD6, NMCS 1, NMCS 2, NMCS 3, NMSPS 2, NMSPS 3, NMSPS 10, NMTPS 1 and NMTPS 9 showed the polymorphic pattern in mango scion/rootstock combinations. The average number of alleles per locus (An), major allele frequency (Maf), gene diversity (GD), expected heterozygosity (Ho) and the polymorphic information content (PIC) of 13 carbohydrate-metabolism specific markers were 2.53, 0.56, 0.52, 0.18 and 0.44, respectively. Further, scion/rootstocks interaction studies showed that different scion/rootstock combinations clustered into one major cluster A, and one outgroup B. Maximum number of scion/rootstocks combination presented in cluster A (80%). 20% scion/rootstock combinations were present in cluster B. Cluster analysis grouped the mango genotypes according to rootstock mostly scion grafted on Kurukkan rootstock were clustered together. It indicates that carbohydrate genes showed the same trend in all scions which were grafted on Kurukkan rootstock and possibly Kurukkan could be utilized to impart regularity in alternate-bearing varieties. Earlier it was reported that the participation of systemic mobile mRNAs in the development of roots, apical meristem, leaves, and underground parts in various plant species involves their long-distance transport between organs, enabled by fellow cells and sieve elements of the phloem system [67, 68]. Furthermore, it was established earlier that numerous mRNAs and non-coding RNAs have been revealed to transfer from the source into sink organs in response to various abiotic and biotic stresses by using grafting [69]. From our results, it was confirmed that variations in all studied traits detected in the leaves and, buds and molecular analysis also explained variations of all graft combinations. The movement of some biochemical constituents may be triggered, while the movement of others might be inhibited in a particular scion-rootstock combination in mango. Further validation is required to ascertain the mobility of metabolites (required for crop load and regularity) from rootstock to scion in mango using mRNAs studied on scion-rootstock combinations using contrasting scion (regular/irregular varieties).

## Conclusion

Based on the results it can be inferred that the physiochemical and nutrient responses of mango scion varieties are manipulated by the rootstock since both scion varieties on different polyembryonic and monoembryonic rootstocks had different biochemical constituents and nutrient content. In addition, we found differential status in starch, sugar, protein content, and C/N ratio in the leaves and reproductive buds of the regular and alternative bearer mango varieties even on similar rootstock. Rootstock Kurukkan enhanced the C/N ratio in both leaves and reproductive buds of the alternate bearing variety ‘Dashehari’ and exhibited content equal to the regular bearer variety. Hence, rootstocks could be able to alter the bearing habit of scion varieties in mango. Furthermore, the reducing sugar and C/N ratio appeared to be rootstock-dependent, however, absorption of most of the nutrients, starch content, and physiological traits seems to be inter-depended. Molecular profiling clearly indicated that Dashehari grafted on Kurukkan rootstock showed more similarity with Amrapali grafted on Kurukkan rootstock, hence both biochemical and molecular data suggested that rootstock could alter the bearing tendency and polyembryonic rootstock Kurukkan was able to modify the bearing habit of alternate bearer ‘Dashehari’. We concluded that in the scion-rootstock combination, an important consideration in mango is to consider for selecting suitable rootstock for alternate/irregular variety and Kurukkan can be utilized as a potential rootstock for modification in bearing habit of alternate bearing varieties in mango.

## Supporting information

S1 Fig Bud images of Amrapali and Dashehari grafted on Kurukkan, Olour and Non-descriptive seedling. (A) Amrapali/Kurukkan, (B) Amrapali/Olour, (C) Amrapali/NDS (D) Dashehari/Kurukkan (E) Dashehari/Olour (F) Dashehari/NDS.
(TIF)

S2 Fig Principal Component analysis biplot of leaf physiological and nutritional traits of scion/rootstock combinations of alternate and regular bearer genotypes of mango. (TIF)

S3 Fig Principal Component analysis biplot of bud physiological and nutritional traits of scion/rootstock combinations of alternate and regular bearer genotypes of mango. (TIF)

S4 Fig SSR profile of NMAD1 in 15 scion/ rootstock combinations of mango. L- 100 bp ladder, 1. Amrapali/Kurukkan, 2. Dashehari/Kurukkan, 3. Mallika/Kurukkan, 4. Pusa Arunima/Kurukkan,5. Pusa Surya/Kurukkan, 6. Amrapali/Olour, 7. Dashehari/Olour, 8. Mallika/Olour, 9. Pusa Arunima/Olour, 10. Pusa Surya/Olour, 11. Amrapali/NDS, 12. Dashehari/NDS, 13. Mallika/NDS, 14. Pusa Arunima/NDS, L- 100 bp ladder, 15. Pusa Surya/NDS. (NDS-non descriptive seedling).
(TIF)

S1 Table Details of carbohydrate metabolism specific genes used for primer designing. (DOCX)

S2 Table List of primer sequences, annealing temperature and product size of carbohydrate metabolism specific primers. (DOCX)
