# Supplementary figures and images for: Rootstock-mediated carbohydrate metabolism, nutrient contents, and physiological modifications in regular and alternate mango (Mangifera indica L.) scion varieties
Source: PLoS One. 2023 May 3;18(5):e0284910. doi: 10.1371/journal.pone.0284910 (PMC10155985; doi:10.1371/journal.pone.0284910)

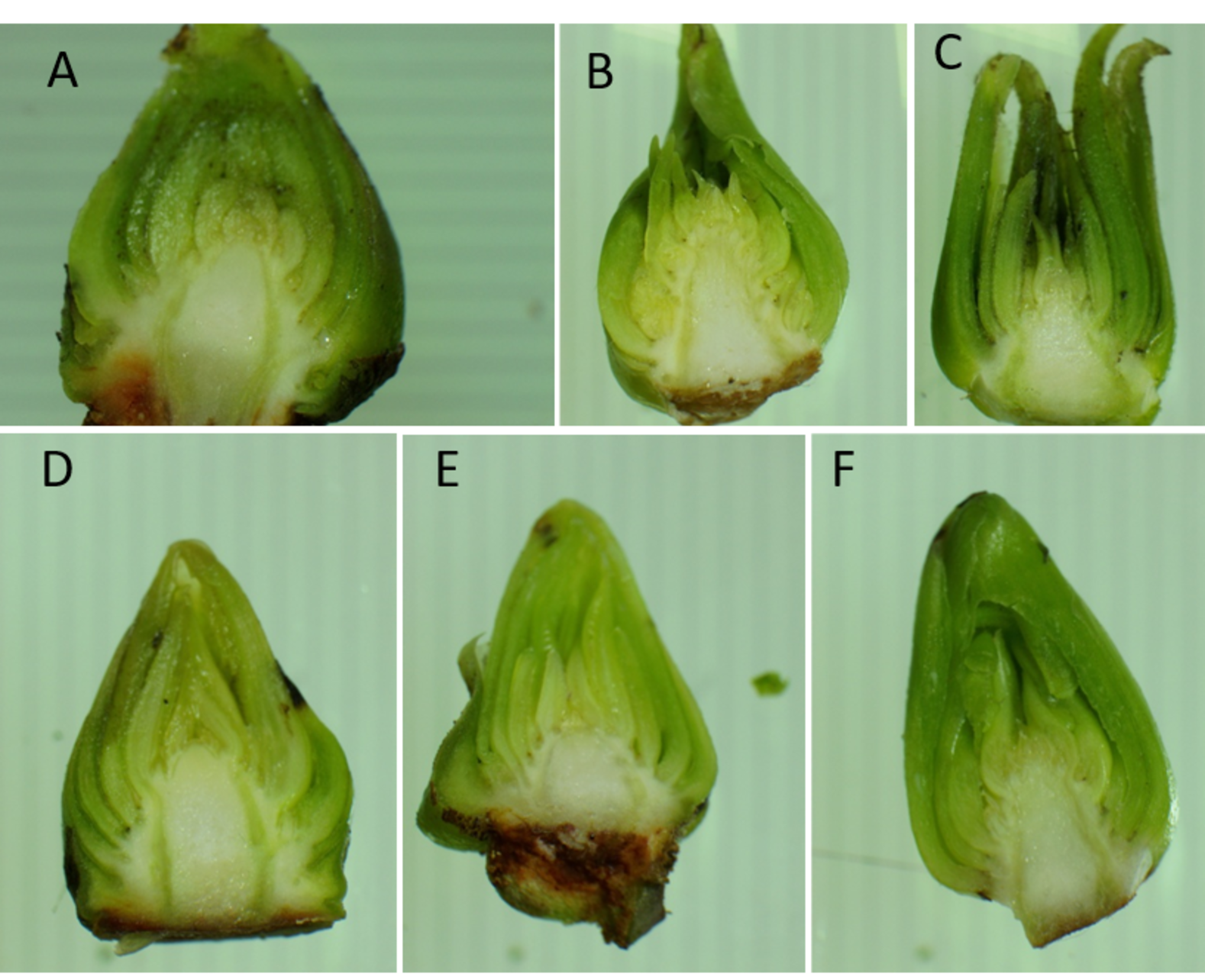

Supplement: S1 Fig — (A) Amrapali/Kurukkan, (B) Amrapali/Olour, (C) Amrapali/NDS (D) Dashehari/Kurukkan (E) Dashehari/Olour (F) Dashehari/NDS. (TIF) [file pone.0284910.s001.tif]

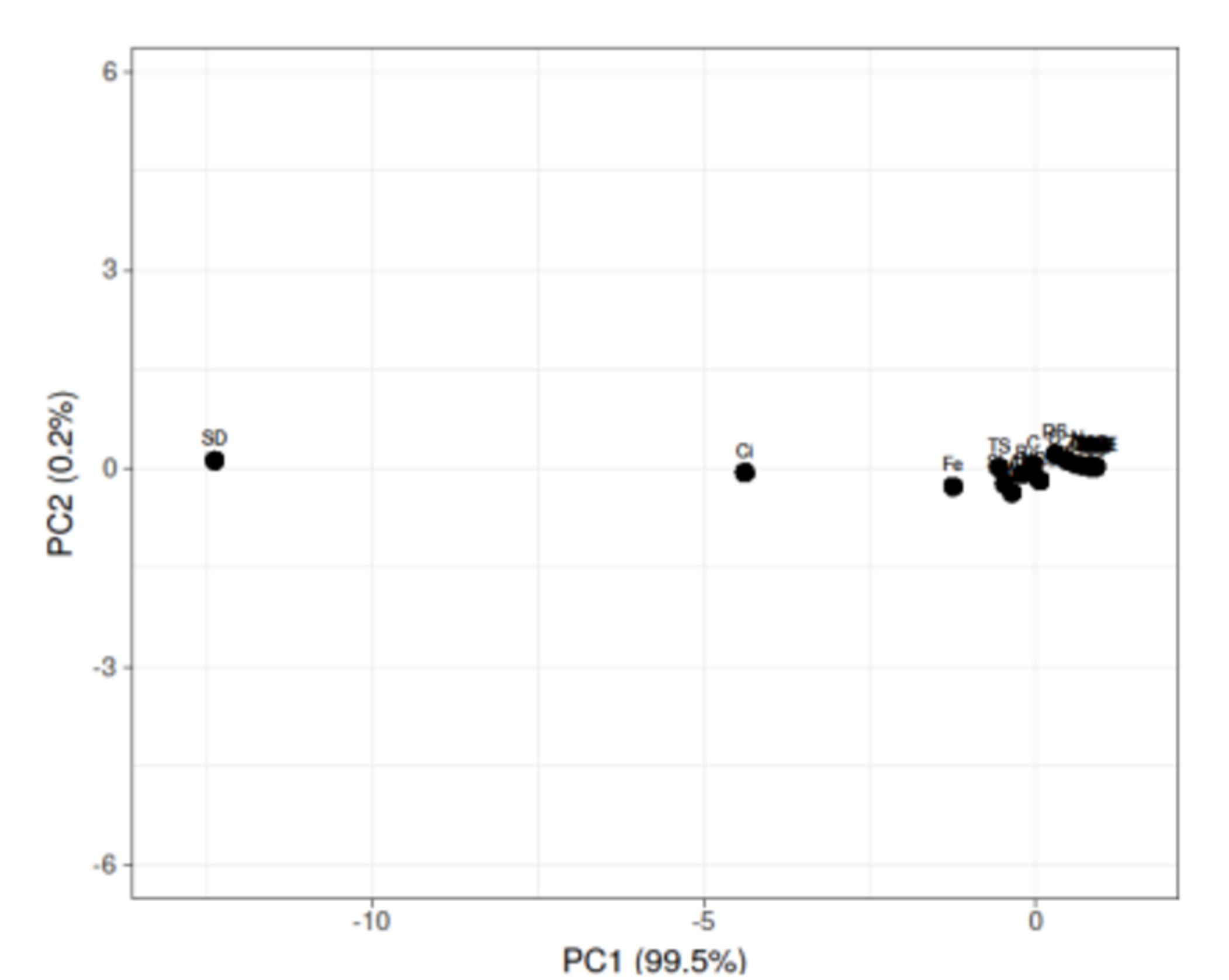

Supplement: S2 Fig — (TIF) [file pone.0284910.s002.tif]

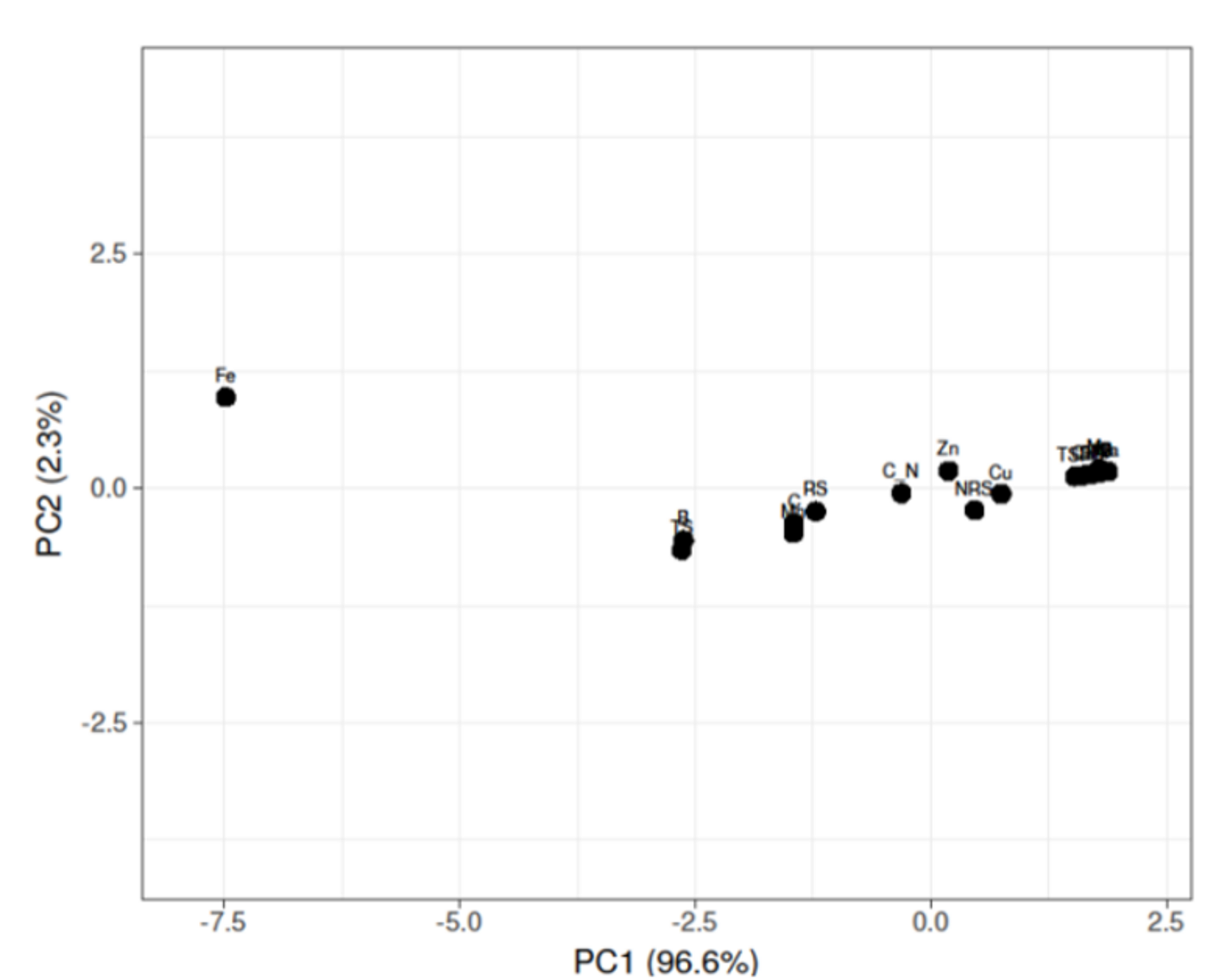

Supplement: S3 Fig — (TIF) [file pone.0284910.s003.tif]

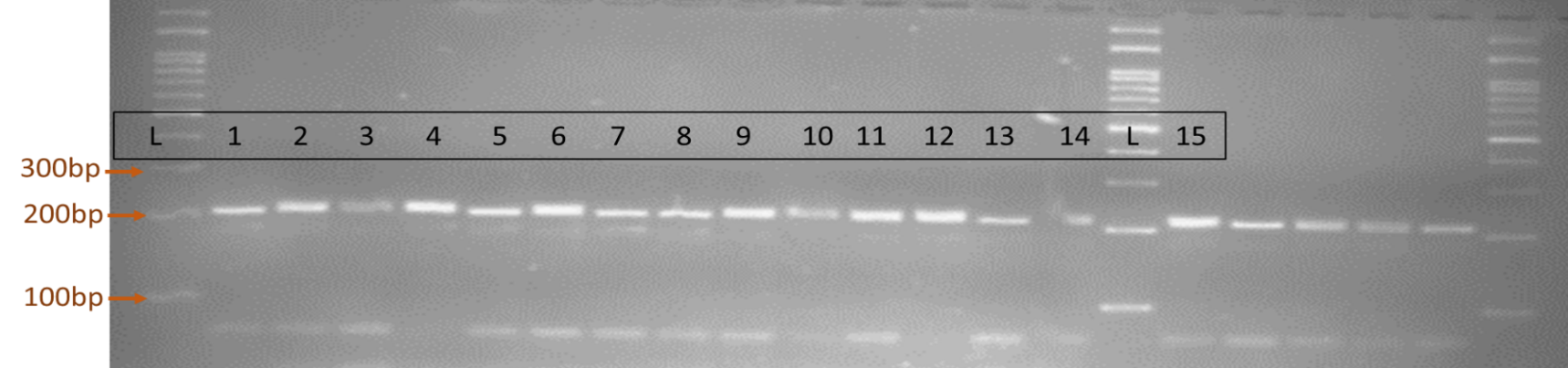

Supplement: S4 Fig — L- 100 bp ladder, 1. Amrapali/Kurukkan, 2. Dashehari/Kurukkan, 3. Mallika/Kurukkan, 4. Pusa Arunima/Kurukkan,5. Pusa Surya/Kurukkan, 6. Amrapali/Olour, 7. Dashehari/Olour, 8. Mallika/Olour, 9. Pusa Arunima/Olour, 10. Pusa Surya/Olour, 11. Amrapali/NDS, 12. Dashehari/NDS, 13. Mallika/NDS, 14. Pusa Arunima/NDS, L- 100 bp ladder, 15. Pusa Surya/NDS. (NDS-non descriptive seedling). (TIF) [file pone.0284910.s004.tif]
