# Supplementary material for: Rootstock-mediated carbohydrate metabolism, nutrient contents, and physiological modifications in regular and alternate mango (Mangifera indica L.) scion varieties
Source: PLoS One. 2023 May 3;18(5):e0284910. doi: 10.1371/journal.pone.0284910 (PMC10155985; doi:10.1371/journal.pone.0284910)
Supplement: S1 Table — (DOCX) [file pone.0284910.s005.docx]

**Table S1. Details of carbohydrate metabolism specific genes used for primer designing.**

| Gene | Protein Accession Number | Nucleotide  Accession Number | Sequence size (bp) | SSRs | Primers |
| --- | --- | --- | --- | --- | --- |
| *Trehalose phosphate synthase* | QCC72920 | MH759789 | 2773 | 13 | 9(NMTPS1-NMTPS9) |
| *Citrate synthase* | AEQ30066,XP_044465751,XP_044465264 | JN001196,XM_044609816,XM_044609329 | 1738 | 5 | 5(NMCS1-NMCS5) |
| *Alcohol dehydrogenase* | ADB43618.1, ADB43617.1, ADB43616.1 | GU233771,GU233770,GU233769 | 4002 | 10 | 6(NMAD1-NMAD 6) |
| *Sucrose phosphate synthase* | BAM68538,BAM68537,BAM68536,BAM68535 | AB724402, AB724401,AB724400,AB724399 | 6970 | 25 | 10 (NMSPS1- NMSPS10) |
