# Supplementary material for: Rootstock-mediated carbohydrate metabolism, nutrient contents, and physiological modifications in regular and alternate mango (Mangifera indica L.) scion varieties
Source: PLoS One. 2023 May 3;18(5):e0284910. doi: 10.1371/journal.pone.0284910 (PMC10155985; doi:10.1371/journal.pone.0284910)
Supplement: S2 Table — (DOCX) [file pone.0284910.s006.docx]

**Table S2. List of primer sequences, annealing temperature and product size of carbohydrate metabolism specific primers.**

| **S.NO.** | **Primer** | **Forward Primer** | **F-TM (°C)** | **Reverse Primer** | **R-TM (°C)** | **Annealing temperature (Ta) (°C)** | **Expected Product size (bp)** |
| --- | --- | --- | --- | --- | --- | --- | --- |
| 1 | NMAD1 | TTTCCATGTTCAGCAGTTTGATT | 53.5 | ACAAAACCATCATCCCACAACA | 55.2 | 55.0 | 218 |
| 2 | NMAD2 | TGCCTGGAGTATACAAGTAGGT | 54.9 | TGAATAACCAGTGGCTTCCC | 54.8 | 55.0 | 241 |
| 3 | NMAD3 | ACAACCAGGAGACCATGTTCT | 56.0 | GCAGGGTTGATCTTGGCAAG | 56.7 | 55.0 | 243 |
| 4 | NMAD4 | CGGTGTTGATTTGAACCCAGT | 55.7 | GCCAAACTTCTTAGCCTGCA | 55.8 | 55.0 | 239 |
| 5 | NMAD5 | TTGATCGGAGCGTTGAATGC | 56.0 | CCCCAACCCTGAGAAGCATA | 56.8 | 55.0 | 160 |
| 6 | NMAD6 | ATTCAAGAAGTGGAGGTGGC | 55.1 | GCTCCTAATTTTCAGCAATGCA | 54.1 | 55.0 | 175 |
| 7 | NMCS1 | GCAACCATGGTGTTCTTCAG | 54.3 | TTTCTTCAGGCGTTCCTGTT | 54.6 | 55.0 | 177 |
| 8 | NMCS2 | CCCTTCAGGTCCAAAGTGAA | 54.7 | CATCTTGGGGTCATCGAATC | 53.1 | 55.0 | 230 |
| 9 | NMCS3 | CACATGTTGGGATTCGATGA | 52.7 | AGCCAAACCATGAAGTGGTC | 55.6 | 55.0 | 189 |
| 10 | NMCS4 | TGGAGTCTTGCGTCAAACAG | 55.3 | CAATCCAAAATGGTTCAGCA | 51.5 | 55.0 | 202 |
| 11 | NMCS5 | AAGCGTAGGGTTTGGTGATG | 55.3 | CACTGCTTTGTTCATTTTTCTCA | 52.3 | 55.0 | 197 |
| 12 | NMSPS1 | TTCTTTCTTCCAGAAGAGTTTAGCA | 54.2 | ACGAGCGATGGAGATCAGTC | 56.5 | 55.0 | 219 |
| 13 | NMSPS2 | GGCATGAACTCAACTTGGTG | 54.3 | AGCCATCATGCATAGCAGAA | 54.4 | 55.0 | 222 |
| 14 | NMSPS3 | TTGATGGTGCTCTCAACCAC | 55.2 | TGAGTGGCCAGTAAAAAGCA | 54.7 | 55.0 | 173 |
| 15 | NMSPS4 | GGTTGCTATGCAATTCTGTCC | 54.2 | CCCTTGATGGTTTTTGTGCT | 53.8 | 55.0 | 225 |
| 16 | NMSPS5 | TTGCCGTGTACATTAGACATCTG | 54.9 | AAAGCACGATGCCAGGTAAT | 54.8 | 55.0 | 220 |
| 17 | NMSPS6 | TTTCTCTTGGCCTGAGCATT | 54.5 | CCCTTTTCTCCATCCAATGA | 52.3 | 55.0 | 186 |
| 18 | NMSPS7 | ACCATTCTGTCAGGCAGTCC | 57.1 | CCAGCACAAACACCTGTTGA | 55.9 | 55.0 | 220 |
| 19 | NMSPS8 | TTGGAGGATTTGGAACTTGG | 52.7 | CCATCCTCTGAGCTGTCTCC | 56.9 | 55.0 | 163 |
| 20 | NMSPS9 | TTTCCTAAATTGCGGGTCAG | 53 | CCTAGCCCCATTCTGACAAA | 54.6 | 55.0 | 183 |
| 21 | NMSPS10 | AGTGGTGGAGCTTAATGACCT | 55.8 | TGAGAGGGTAACTCCGGTTG | 56.2 | 55.0 | 208 |
| 22 | NMTPS1 | GCCACTACCAGGAGTTTCCA | 56.8 | GGTGTGTGGAGAAACCAACC | 56.3 | 55.0 | 191 |
| 23 | NMTPS2 | GCTGCTGATTTGGTTGGTTT | 54.3 | TCTCAAGTGCTCGCATGAAC | 55.4 | 55.0 | 178 |
| 24 | NMTPS3 | TGGATCGCTCTCTGGACTTT | 55.7 | CTCCAGCACCCAAAGATTGT | 55.3 | 55.0 | 186 |
| 25 | NMTPS4 | ACAATCTTTGGGTGCTGGAG | 55.3 | GGAAGATTAGGAGGGGCTTG | 55 | 55.0 | 237 |
| 26 | NMTPS5 | AGCAATCGACCGTATTTTGG | 53.2 | GAGGTTGAGGGCCTTCTCTC | 57.2 | 55.0 | 210 |
| 27 | NMTPS6 | GGCATTTTCTGGGAAAGGAT | 53.1 | ACTGGCACAGTGTTGAGCAG | 58 | 55.0 | 239 |
| 28 | NMTPS7 | AGAAGGCCCTCAACCTCATT | 56.2 | ATTTGTTCGAGTCCGTCCAA | 54.3 | 55.0 | 219 |
| 29 | NMTPS8 | GGGTGAGTGAAGGAGGTGAA | 56.5 | TCCATCTTGCCTCAAACTCC | 54.8 | 55.0 | 180 |
| 30 | NMTPS9 | GGCGAACAACACCAGAAAAG | 54.7 | GCTTGATGATGATGAGGAAGAA | 52.7 | 55.0 | 188 |
